# Population Pharmacokinetic Modeling for Twice-Daily Intravenous Busulfan in a Large Cohort of Pediatric Patients Undergoing Hematopoietic Stem Cell Transplantation—A 10-Year Single-Center Experience

**DOI:** 10.3390/pharmaceutics16010013

**Published:** 2023-12-20

**Authors:** Katharina M. Schreib, Dominic S. Bräm, Ulrike Barbara Zeilhofer, Daniel Müller, Tayfun Güngör, Stefanie D. Krämer, Mathias M. Hauri-Hohl

**Affiliations:** 1 Department of Stem Cell Transplantation, University Children’s Hospital Zurich—Eleonore Foundation & Children’s Research Center (CRC), University of Zurich, 8032 Zurich, Switzerland; katharina.schreib@kispi.uzh.ch (K.M.S.); ulrike.zeilhofer@kispi.uzh.ch (U.B.Z.); tayfun.guengoer@kispi.uzh.ch (T.G.); 2 Institute of Pharmaceutical Sciences, Department of Chemistry and Applied Biosciences, ETH Zurich, 8093 Zurich, Switzerland; dominic.braem@ukbb.ch; 3 Institute for Clinical Chemistry, University Hospital Zurich, 8091 Zurich, Switzerland; daniel.mueller@usb.ch

**Keywords:** intravenous busulfan, pediatric HSCT, pharmacokinetics, PK modeling

## Abstract

Reaching target exposure of busulfan-based conditioning prior to hematopoietic stem cell transplantation is vital for favorable therapy outcomes. Yet, a wide inter-patient and inter-occasion variability in busulfan exposure has been reported, especially in children. We aimed to identify factors associated with the variability of busulfan pharmacokinetics in 124 consecutive patients transplanted at the University Children’s Hospital Zurich between October 2010 and February 2020. Clinical data and busulfan plasma levels after twice-daily intravenous administration were analyzed retrospectively by population pharmacokinetic modeling. The volume of distribution correlated with total body water. The elimination rate constant followed an age-dependent maturation function, as previously suggested, and correlated with the levels of serum albumin. Acute lymphoblastic leukemia reduced busulfan clearance by 20%. Clearance significantly decreased by 17% on average from the start to the third day of busulfan administration, in agreement with other studies. An average reduction of 31% was found in patients with hemophagocytic lymphohistiocytosis and X-linked lymphoproliferative disease. In conclusion, we demonstrate that in addition to known factors, underlying disease and serum albumin significantly impact busulfan pharmacokinetics in pediatric patients; yet, substantial unexplained variability in some patients remained. Thus, we consider repeated pharmacokinetic assessment essential to achieve the desired target exposure in twice-daily busulfan administration.

## 1. Introduction

Allogeneic hematopoietic stem cell transplantation (HSCT) is an established therapy for malignant and non-malignant disorders of the hematopoietic system. Busulfan, which mainly targets hematopoietic stem and progenitor cells, remains the backbone of many myeloablative conditioning regimens for both autologous and allogeneic HSCT [1,2,3]. Oral dosing of busulfan is complicated by its high variability in oral bioavailability. Intravenously administered, it not only demonstrates better tolerability but also reduces inter-patient variability of pharmacokinetics (PK) [4,5].

Assessment of individual PK is critical, as both intended and adverse biological effects of busulfan correlate with the cumulative exposure assessed by the area under the plasma concentration-time curve (AUC) or concentration at a steady-state [6,7]. Overexposure to busulfan correlates with higher toxicity (e.g., sinusoidal obstruction syndrome (SOS) and mucositis), increased risk of acute graft vs. host disease, and transplantation-related mortality [8,9], while underexposure is associated with increased incidence of graft failure and disease relapse [10,11,12]. The optimal level of cumulative busulfan exposure (cAUC) is still under debate. Bartelink and colleagues recently demonstrated an association of a cAUC in the range of 78–101 mg · h/L with superior event-free survival for patients with malignant and non-malignant diseases [13]. Yet, submyeloablative cAUCs (between 45–65 mg · h/L) were sufficient for excellent results in primary immunodeficiencies [11,14,15,16,17].

Generally, multiple doses of busulfan are required to reach the target exposure. The starting dose is either established by weight-based nomograms or extrapolated from the PK of a pre-conditioning test dose [18,19,20,21]. Although improved outcome was associated with lower AUC of the first dose [17], it is generally accepted that cAUC is relevant for the safety and efficacy of busulfan [13]. Also, in addition to significant inter-patient variability, inter-occasion alterations of busulfan PK argue for repeated AUC assessment of the individual doses, especially in pediatric patients [7,11,18,22]. Population PK models based on patient data allowed the establishment of improved dosing predictions [18,23,24]. Nevertheless, a substantial fraction of patients elude accurate prediction of the AUC of the first and subsequent doses, which is evidence of substantial between-subject and -occasion variability. The large inter-patient variability of busulfan PK, especially in pediatric patients, is influenced by various factors including age, body weight, disease, drug interactions, and hepatic metabolism [18,25,26,27,28,29,30]. In addition, as a large fraction of busulfan irreversibly binds to leukocytes, erythrocytes, and plasma proteins (mainly albumin) [31,32], these patient-intrinsic factors should be considered, as they may contribute to busulfan elimination.

The conjugation of busulfan to glutathione (GSH) mediated by particular isoforms of the glutathione-S-transferase (GST) results in its inactivation. Inter-individual variability in hepatic busulfan clearance (CL) is influenced by differential expression levels of GST, as well as by the abundance of GSH, which is depleted by busulfan and other drugs [28,33,34,35,36]. N-acetylcysteine (NAC), the precursor of GSH, repletes intrahepatic GSH levels and may thus aid in the prevention of busulfan-induced liver toxicity seemingly without interfering with the desired myeloablative effects of busulfan [37].

In this single-center study, we interrogated the effects of patient factors, co-medications, and laboratory parameters with potential biological significance on busulfan clearance of the first and subsequent doses. Strict twice-daily administration of busulfan in the whole primarily pediatric cohort, a high number of plasma concentration measurements of successive doses in individual patients, and the incorporation of biologically relevant data allowed for the identification of factors contributing to inter-patient and inter-occasion variability of busulfan clearance.

## 2. Methods

### 2.1. Study Design and Patients

In this retrospective single-center study, we analyzed PK and clinical data from 124 consecutive patients undergoing allogeneic or autologous HSCT after a strict twice-daily busulfan-based conditioning regimen between October 2010 and February 2020 at the University Children’s Hospital Zurich, Switzerland.

### 2.2. Busulfan Treatment Regimen

The initial busulfan dose was calculated using recommended weight-based dosing nomograms. Between October 2010 and January 2016, the European Medicines Agency recommendation for weight-based busulfan dosing was used [19], while from January 2016 to February 2020, the individualized dosing table by Bartelink et. al. [18] was applied. For the twice-daily busulfan dosing used in our study, the recommended daily dose was divided into two doses [18,19].

Busulfan was administered twice-daily (starting at 4 or 5 a.m./p.m.) as an intravenous infusion over either four hours (from October 2010 to September 2014) or three hours (from October 2014 to February 2020; Figure S1). The initial dose was intended to target an exposure, expressed by the AUC, of 9–12 mg · h/L according to Bartelink et al. [13]. Patients with non-malignant diseases (except patients with metabolic diseases) received a reduced intensity conditioning (RIC) regimen, targeting a cumulative exposure (expressed as cAUC) of 45–70 mg · h/L [14,15]. Patients with malignant and metabolic diseases were targeted with a cAUC of 80–100 mg · h/L, which was considered myeloablative conditioning (MAC) [13]. To achieve the target cumulative exposure, patients received between four and ten doses over two to five consecutive days.

### 2.3. Co-Medication

Figure S2 provides an overview of the co-medication. Prior to the start of conditioning, oral amphotericin B, gentamicin, vancomycin, and polymyxin B were given for gut decontamination. Ondansetron was administered daily to prevent and treat chemotherapy-induced nausea. All patients received mainly clonazepam or rarely levetiracetam as prophylaxis for busulfan-associated seizures. A high dose of NAC (200 mg/kg BW) was given repeatedly to most of our patients (69%) as a once- or twice-daily infusion (2–3 h) with the intent to mitigate busulfan-mediated liver injury. Paracetamol and corticosteroids were given as co-medications of serotherapy (alemtuzumab or anti-thymocyte globulin, Figure S2), while paracetamol was also used as a common analgesic and antipyretic agent.

### 2.4. Busulfan Sampling and Dose Adjustments

Busulfan plasma concentrations (*C*(*t*)) were measured after the morning infusion. Heparinized blood samples were taken right before drug administration and at 0, 30, 60, 120, 240, and 360 min after the end of the respective infusion. The plasma was immediately separated from blood cells by centrifugation and stored at −80 °C until further analysis on the day of sampling. *C*(*t*) were measured after protein precipitation with methanol at the Institute for Clinical Chemistry at the University Hospital of Zurich by liquid-chromatography coupled to tandem mass spectrometry (LC-MS/MS). The method was fully validated and accredited according to ISO 17025.

Subsequent busulfan doses were, if necessary, adjusted based on calculated exposure expressed as the AUC to reach the previously mentioned target cAUC. Repeated TDM was performed based on the physician’s decision (Figure S1).

### 2.5. Population Pharmacokinetic Analysis

Population PK modeling was performed with an open one-compartment model with infusion, using the open-source software environment R (R-project, version 4.2.0) with the saemix package (version 3.0) for non-linear mixed effects modeling [38,39]. The saemix package builds on stochastic approximation expectation maximization [40]. The R-scripts of this study are deposited on gitlab.ethz.ch (https://gitlab.ethz.ch/skraemer/busulfan_2022.git, accessed on 17 December 2023). Fit PK parameters were the natural logarithm (ln) of the volume of distribution (*V*) and the ln of the elimination rate constant (*k*). Two additional parameters (*d_k_* and ln(*κ_k_*)) were introduced to account for a change in *k* from the first to subsequent dosing intervals. Parameter *d_k_* is the amplitude of the exponential function describing the difference between *k* at time 0 and hypothetical *k* at infinity. Parameter *κ_k_* is the exponent of this exponential change. ln(2)/*κ_k_* is thus the half-life of the change in *k*. The fit function for an individual patient with *N* dosing intervals, before including covariates and random effects, is shown in Equation (1).

(1) $$C\left(t\right)=\sum_{j=1}^{N}\frac{R_{0,j}}{k^{\prime }\left(t\right)\times V}\times (\left(1-exp\left(-k^{\prime }\left(t\right)\times t_{inf}\right)\right)\times \left(t_{inf}\leq T_{inf,j}\right)+(1-exp\left(-k^{\prime }\left(t\right)\times T_{inf,j}\right))\times \;\left(\left(T_{inf,j}+t_{0,j}\right)<t\right))\times exp\left(-k^{\prime }\left(t\right)\times t_{el}\right)$$

(2) $$k^{*}\left(t\right)=k\times \left(-d_{k}\times \left(exp\left(-k_{k}\times t_{mid}\right)-1\right)+1\right)$$

(3) $$k^{\prime }\left(t\right)=\left(\left(1+d_{k}\right)\times k\times t-\frac{d_{k}\times k+{(k}^{*}\left(t\right)-(1+d_{k})\times k)}{{Κ}_{k}}\right)/t$$

(4) $$t_{inf}=\left(t-t_{0,j}\right)\times (\left(t-t_{0,j}\right)\leq T_{inf,j})\times (\left(t-t_{0,j}\right)\geq 0)$$

(5) $$t_{el}=\left(t-t_{0,j}-T_{inf,j}\right)\times \left(\left(t-t_{0,j}-T_{inf,j}\right)\geq 0\right)$$

In Equations (1)–(5), *t* denotes time, *R*_0,*j*_ is the infusion rate in mass/*t* of the *j*-th infusion, *k*^∗^(*t*) is the time-dependent *k* as calculated with the mid-time point (*t*_mid_) of the respective dosing interval (Equation (2)), and *k*′(*t*) is the average rate constant at the individual dosing interval (Equation (3)), as calculated from the integral of *k*^∗^(*t*). *t*_inf_ is the time during infusion (Equation (4)), *T*_inf,*j*_ is the infusion duration of the *j*-th infusion (3 h or 4 h), *t*_0,*j*_ is the start time of the *j*-th infusion, and *t_e_*_l_ is the time after infusion stop (Equation (5)). In Equations (1)–(5), terms with logical expressions equal 1 if true and 0 if false. Additional details on the calculation of *k*′(*t*) are provided in the *R* script kchange.R on gitlab.ethz.ch.

Random effects were included on patients’ levels as described under Section 2.7 and in the Section 3. In all tested models, the error model was “additive”, i.e., a constant value, as described in more detail by the authors of the saemix package [39]. Applying a proportional error model (in the absence of covariates and before including *d_k_* and ln(*κ_k_*) as fit parameters) failed to compute -2LL and resulted in an asymmetrical distribution of the residuals (predicted *C*(*t*) at the subject level minus observed *C*(*t*)). Alternative error models were not tested, considering the relatively narrow range of the observed concentrations (Figure S3). Initial values for ln(*V*) and ln(*k*) were randomly sampled from varying ranges around values estimated from individual fits of *C*(*t*) vs. *t* of the first dosing interval (2.5 for ln(*V*) and −1 for ln(*k*) with *V* in L and *k* in 1/h). The ranges for the final model were ±1 of these values. The start values for *d_k_* and ln(*κ_k_*) were 0 and −3.5, respectively. An ln(*κ_k_*) of −3.5 corresponds to a 24 h half-life of the change in *k* (ln(ln(2)/24 h) = −3.5). In the final model, the number of iterations set in the options of saemix (nbiter.saemix) were 2000 for the exploration phase and 300 for the smoothing phase. For model building, they were 600 and 100, respectively. Higher numbers had no significant effects on the results.

### 2.6. Non-parametric Estimation of AUC, C_max_, CL, V, t_1/2_, and MRT

One-compartment model elimination rate constants *k_ij_* were calculated for each individual patient (*i*) and individual dosing interval (with measurements of plasma concentrations) (*j*) as the negative slope of the linear regression of the respective descending phase of ln(*C*(*t_ij_*)) vs. *t_ij_*. The respective AUC*_ij_* was estimated with the trapezoidal method. The AUC of the first dosing interval (AUC*_i_*_1_) was calculated as the sum of the trapezoids up to *t_n_*, where *t_n_* is *t* at the last *C*(*t*) measurement of the 1st dosing interval plus the extrapolated area from *t_n_* to infinity. The latter was calculated as *C*(*t*) of the last measurement before the second infusion (*C*(*t_n_*)) divided by the minus slope of the last 3 ln(*C*(*t*)) vs. *t* of the first dosing interval (*k_n_*). *C*(*t_n_*)/*k_n_* is the integral of *C*(*t_n_*) × exp(−*k*_n_ × (*t* − *t_n_*)). The AUC*_ij_* of subsequent dosing intervals was calculated from the trapezoids between two infusion starts assuming that a steady state was reached (no corrections for residual areas). Missing *C*(*t*) before the next infusion start were extrapolated from the last 3 *C*(*t*) measurements of the respective dosing interval (linear extrapolation of ln(*C*(*t*)) ~ *t* to *t* of the start of the second infusion). C_max,*ij*_ was defined as the maximal observed *C*(*t*) of a dosing interval. CL*_ij_* was calculated as dose*_ij_*/AUC*_ij_* and *V_ij_* as dose*_ij_*/(AUC*_ij_* × *k_ij_*). The elimination half-life *t*_1/2,*ij*_ was calculated as ln(2)/*k_ij_* and the mean residence time (MRT*_ij_*) as AUMC*_ij_*/AUC*_ij_*, where AUMC*_ij_* is calculated similarly to the AUC*_ij_* but with *C*(*t*) × *t* replacing *C*(*t*) and *C*(*t_n_*) × *t/k_n_* + *C*(*t_n_*)/(*k_n_*)^2^ as the extrapolated AUMC from *t_n_* to infinity [41].

### 2.7. Covariate Analysis

For model building, we followed the strategies presented by [42,43]. To identify the basic model, we started without any covariate and then included individual parameters as potential covariates. Potential covariates were identified by plotting the random effects of each PK parameter as well as the non-parametric PK parameters against all available patient-specific data. These included several body size metrics such as body weight (*W*), body height (*H*), calculated body surface area (BSA, Equation (6); [44]), calculated total body water (TBW, Equation (7), [45]), and calculated fat-free mass (FFM, Equation (8), [23]). Patient-specific data furthermore included age and an age-dependent maturation function for busulfan CL (*F*_mat_, Equation (9); [23]), for which postmenstrual age (PMA) was calculated by adding a fixed value of 40 weeks to the patient’s postnatal age. They furthermore included sex (defined by external sex characteristics) and underlying disease. Depending on their condition requiring HSCT, patients were allocated to nine disease groups (acute lymphoblastic leukemia, ALL; acute myeloid leukemia, AML; chronic granulomatous disease; hemoglobinopathies; hemophagocytic lymphohistiocytosis/X-linked lymphoproliferative disease, HLH/XLP; primary immunodeficiencies; metabolic diseases; neuroblastoma; and others; as shown in Table 1) and these were tested as potential covariates. We investigated the potential influence of plasma protein levels (total protein and serum albumin), hematocrit, and white blood cell (leukocytes) count on busulfan PK. We evaluated treatment-related factors, namely whether *C*(*t*) measurements were from the uneven or even dose numbers (whether the first busulfan dose was in the morning or evening, with PK measurements performed only after the morning infusions), busulfan dosing schemes [18,19], and conditioning regimen (RIC vs. MAC), and whether *T*_inf_ was 3 h or 4 h. When a value of a covariate was missing, it was set to the respective reference value used in the model. Reference values were chosen close to their respective median in the studied population (see Section 3). We finally searched for potential effects of co-medication administered within 24 h before the respective busulfan infusion (plotting the difference between observed and predicted *C*(*t*) (residues) against co-medication, as well as plotting the observed vs. predicted *C*(*t*)). We chose a time frame of 24 h, as the *t*_1/2_ of most drugs is shorter than that, resulting in a low or negligible effect after a longer time period.

(6) $$BSA\left[m^{2}\right]={\left(H\left[cm\right]\times W\left[kg\right]/3600\right)}^{0.5}$$

(7) $$TBW\left[L\right]=exp(-2.952+0.551\times ln\left(W\left[kg\right]\right)+0.796\times ln\left(H\left[cm\right]\right)\;+0.008\times age\left[y\right]+female\times \left(-0.047\right))$$

(8) $$\begin{array}{l} FFM\left[kg\right]\\ =\left(female\times 37.99+male\times 42.92\right)\times {\left(H\left[m\right]\right)}^{2}\\ \times W\left[kg\right]/\left(\left(female\times 35.98+male\times 30.93\right)\times {\left(H\left[m\right]\right)}^{2}+W\left[kg\right]\right) \end{array}$$

(9) $$F_{mat}=1/\left(1+{\left(PMA\left[weeks\right]/46\right)}^{-2.3}\right)$$

Terms in square brackets in Equations (6)–(9) indicate the unit of the respective value. The terms female and male are 1 if true and 0 if false.

In each search round for covariates, the covariate that reduced −2 × ln(likelihood by important sampling) (-2LL) the most was kept in the model as long as the reduction was larger than 3.84, i.e., the critical value of *χ*^2^ at 1 − *p* with *p* = 0.05 at one degree of freedom (df) difference. Covariates were excluded if their maximal effect in the final model was small (comparing the effect at the lowest and highest covariate value in the population), namely between −0.1 and +0.1, or if their *p*-value was >0.05. All included covariates were subsequently evaluated individually by backward elimination. Covariates were kept in the model if their elimination increased the -2LL by more than 6.63, i.e., the critical value of *χ*^2^ at 1 − *p* with *p* = 0.01. Comparing two models with an equal number of fit parameters (equal df), the model with the lower -2LL was preferred. Random effects were included if the respective shrinkage as calculated from the respective saemix function as 1—variance (subject predicted parameter—population predicted parameter)/variance(predicted inter-individual variability) was lower than 0.4 in the basic model [46].

As co-medications varied during the therapy of an individual patient (Figure S2), we included the effects as fit parameters in the objective function rather than as covariates. During co-medication (within 24 h before infusion start), the test ln(PK parameter) in the objective function was adjusted to ln(PK parameter) + ln(effect of drug).

### 2.8. Simulations of CL with Time

To simulate the relative change in the CL over *t* (CL(*t*)/CL), which is equivalent to *k*′(*t*)/*k,* as *V* did not change with *t* and CL = *k* × *V*, a series of *k*′(*t*)/*k* were calculated according to Equations (2) and (3) as *d_k_* × exp(−*κ_k_* × *t*) from a set of 100,000 randomly sampled values in a normal distribution of the fit *d_k_*, with SD as the square root of the fit variance of the random effects of *d_k_*. For *κ_k_*, the 100,000 values were randomly sampled from a normal distribution of the fit *κ_k_* where the SD of the normal distribution equalled the fit SE of *κ_k_* multiplied by the square root of the number of subjects (as no random effects were fit for *κ_k_*).

### 2.9. Model Evaluation

The final model was evaluated by plotting the observed vs. the predicted *C(t)*, inspecting the distribution of the individual weighted residuals obtained with the conditional estimates (conditional individual weighted residuals, icwres) and of the normalized prediction distribution errors (npde), a visual predictive check (vpc) plot, the distribution of the fit parameters, the reproducibility of the results, analysis of resampled data (bootstrapping), and splitting the data into a training and a test set.

The icwres and npde (10,000 simulations) were calculated with the respective functions in the saemix package. The vpc plot was generated with the median and 5 and 95% percentiles of the observed *C*(*t*) and simulated *C*(*t*) using the saemix function simul.saemix with the fit saemix object of the final model for 1000 simulations.

For the bootstrapping, resampled datasets had the same size as the original dataset, allowing subjects to be present multiple times in a resampled dataset (function sample with attribute replace = TRUE in R). Start values for the analysis of the resampled datasets were 2.5 for ln(*V*), −1 for ln(*k*), ln(ln(2)/24) for ln(*κ_k_*), and 0 for all other parameters with *V* in L, *k*, and *κ_k_* in 1/h (no random sampling of the start values, which is in contrast to the main analysis). The number of iterations were as described under Section 2.5 (Methods) for the final model. Confidence intervals (95%) of the resampled results were defined as the fit parameters at the 2.5 and 97.5% positions of the sorted fit parameters of the resampled datasets.

To simulate an external evaluation of the model, we split the data (R function sample) into a training and a test set with 93 and 31 patients, respectively (ratio 75/25), built the model with the training set, and inspected the predictions for the test set by plotting the observed vs. predicted *C*(*t*) and calculating the sum of squared residues (SSRs) divided by the number of *C*(*t*) values for training and test set.

## 3. Results

### 3.1. Patient Characteristics

The characteristics of the 124 patients included in this study are presented in Table 1. The majority of the patients, namely 118, were younger than 18 years, and 23 were younger than 1 year (postnatal age). The distributions of age, *W*, BSA, plasma albumin, hematocrit, and leukocyte count (Leu) are shown in Figure S1. As mentioned in the Section 2.5, four to ten busulfan doses were required to reach the target cAUC. *C*(*t*) measurements of busulfan were performed for a minimum of one dose and a maximum of five doses per patient (a median of three doses with *C*(*t*) measurements, Figure S1G). In total, 2376 busulfan plasma concentrations from 373 busulfan infusions were measured and used for population PK analysis. No data were excluded from the analysis. Figures S4–S9 show the non-parametric PK parameters plotted against the patient characteristics.

### 3.2. Population Pharmacokinetic Analysis

The key steps in model building for the population PK analysis are described in Table 2. An open one-compartment model is typically defined by two PK parameters, namely *V* together with either *k* or CL. We first evaluated the correlations between the non-parametric parameters CL, *V,* and *k* (Figure S10). Furthermore, we analyzed the data by population PK with both a model based on *V* and *k* and a model based on *V* and CL, without including any covariate. Both analyses (non-parametric and one-compartment model) revealed a stronger relationship between *V* and CL than between *V* and *k*. We chose the model with lower correlation between the fit parameters, namely the model based on *V* and *k*, for further analysis of the data. Building the model on *V* and CL would result in two parameters with similar covariates (as concluded from their correlation), potentially masking the covariates for *k* and consequently for *t*_1/2_ (*t*_1/2_ = ln(2)/*k*) of busulfan in our study.

From both non-parametric analysis and population PK modeling, we observed that both *k* and CL, in general, decreased from the first to subsequent dosing intervals (*t*_1/2_ increased). This is shown for non-parametric *k* and CL in Figures S11 and S12. To account for this change, we replaced the constant *k* in the population PK model with a dynamic parameter, allowing an exponential change in *k* with *t*, as described in Equations (1)–(3). According to Equations (1)–(3), *k*′(t) can only change from one dosing interval to the next but not within a dosing interval. This prevents the model from masking a 2-compartment model by changing *k* within a dosing interval.

Fitting a two-compartment model to the data did not reduce -2LL compared to the above-described one-compartment model with dynamic *k*′(t) without including covariates (Table 2). We, therefore, used the above-described one-compartment model with ln(*V*), ln(*k*), *d_k_*, and ln(*κ_k_*) as fit parameters (Equation (1)) for all subsequent analyses. The data were first analyzed with the above-described basic model (model 3* in Table 2) without including any covariate. The resulting random effects for ln(*V*), ln(*k*), and *d_k_* are plotted against potential covariates in Figures S13–S15. Random effects of ln(*κ_k_*) were omitted to avoid overparameterization, as judged from the shrinkage of the fit parameters. The shrinkage for ln(*κ_k_*) in the basic or final model, if including ln(*κ_k_*) random effects, was >60%, indicating overparameterization (Table 2).

For the plots in Figures S13–S15, numeric (continuous) covariates (*X*) were transformed to Δln(*X*) = ln(*X*/*X*_ref_), where *X*_ref_ is the reference value of *X*, i.e., a rounded value close to the population median of *X*. The respective reference values are shown in Table 3. These plots (Figures S13–S15) revealed strong correlations between Δln(TBW) and the random effects of both ln(*V*) and ln(*k*). Δln(TBW) was included as a covariate for both parameters. Before including additional covariates, the data were inspected for correlations between potential covariates (Figure S16). This facilitated the choice of alternative covariates for comparison, e.g., Δln(TBW) compared to Δln(*W*) or Δln(*BSA*) as a covariate for ln(*V*), as shown in Table 2. Stepwise forward selection and backward elimination of potential covariates according to Table 2 resulted in model 16**, without testing for potential effects of co-medication yet.

The covariate ln(*F*_mat_) for ln(*k*) became apparent and significant after introducing Δln(TBW) as a covariate for ln(*k*), indicating that it is a correction factor for TBW in our model at an age lower than ~1 year with a value of 0.85 at 98 weeks of postmenstrual age (*F*_mat_ is a sigmoidal function from 0 to 1 of postmenstrual age with a value of 0.5 at 46 weeks of postmenstrual age [23]). ln(*F*_mat_) was also significant as a covariate if Δln(*W*) was used instead of Δln(TBW) as a covariate for ln(*k*). Replacing Δln(TBW) as a covariate on ln(*k*) by other body size metrics in model 16** did not further reduce -2LL.

The plots of the random effects vs. potential covariates of model 16** in Table 2 (Figures S17–S19) suggested an effect of ln(hematocrit) on ln(*V*) and of ln(Leu) on ln(*k*). However, neither parameter fulfiled the criteria for being included in the final model (Table 2). Neither sex, conditioning regimen, nor busulfan dosing scheme had an additional significant effect on busulfan PK, as concluded from plotting the random effects of model 16** (Figures S17–S19) or the non-parametric PK parameters (Figures S4–S9) against the patient properties. No significant effect was observed whether the first busulfan dose was administered in the morning or evening.

Regarding co-medication, we compared observed vs. predicted *C*(*t*) from model 16** in the presence and absence of co-medication, as well as predicted minus observed *C*(*t*) with and without co-medication. Potential effects were detected for NAC (underestimation of low *C*(*t*)), clofarabine in the presence of NAC (overestimation of *C*(*t*); however, there were only a few data), and paracetamol in the absence of NAC (underestimation of *C*(*t*)). Figure S20 shows the respective observed vs. predicted *C*(*t*). The figure also includes the data for fludarabine, a drug in discussion for affecting busulfan PK [18,24,33,47,48,49,50,51]. However, none of the administered co-medications reached the criteria for inclusion in the model; either Δ-2LL or the effect (absolute value) was too low (Table 2). The effect of NAC on ln(*V*), increasing *V* by ~10%, was close to reaching the inclusion criteria (Table 2). Whether NAC was co-administered or not had no effect on the parameter *d_k_*, i.e., on the change in *k* (and CL) over time at the subject level (Figure S21). None of the other drugs showed an effect on *C*(*t*). Model 16** in Table 2 was, therefore, considered the final model for busulfan population PK modeling with the available data. The respective fit parameters are shown in Table 3.

While the effect of Δln(TBW) on ln(*V*) was close to 1 in the final model (0.931 in Table 3) and in agreement with a proportional change in *V* with TBW, the effect on ln(*k*) was weaker, with an effect size of −0.189. Doubling the TBW thus results in a reduction in *k* by 12% (factor 2^−0.189^) at a postnatal age > ~1 year where *F*_mat_ becomes close to 1. ALL significantly reduced *k* by 20% on average (100 × (1 − 10^−0.21^), while the reduction in *k* over time was amplified from 17% (−0.167 in Table 3) to 31% (−0.167 + (−0.145)) if the diagnosis belonged to the HLH/XLP group. Furthermore, *k* increased with albumin level with a 6% change in *k* for a 5 g/L change in albumin concentration (for the range 25 to 35 g/L, e.g., (25/30)^0.331^). Parameters *V* and *k* were affected by *T*_inf_. We hypothesize that shortening *T*_inf_ from 4 h to 3 h uncovered a distribution phase in the *C*(*t*) vs. *t* curve, corresponding to a two-compartment model. This would result in a higher apparent *k* at 3 h *T*_inf_ at a correspondingly smaller *V*, as supported by the opposite effect sizes (−0.161 for ln(*k*) and 0.226 for ln(*V*), Table 3). The calculated CL (=*k* × *V*) is thus virtually independent of *T*_inf_.

**Figure 1:**
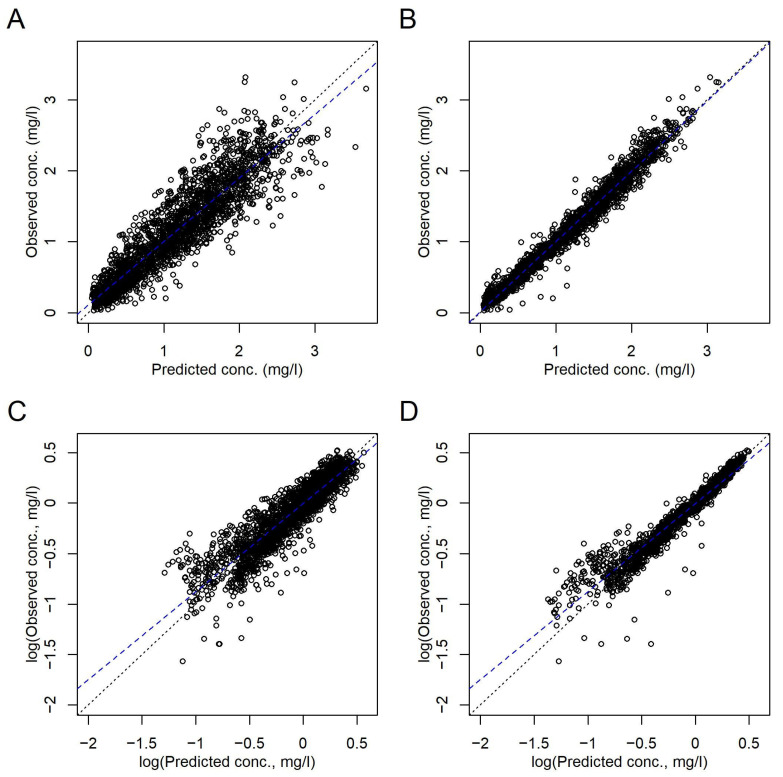
Comparison of the observed and predicted *C*(*t*) according to the final model in Table 3. (**A**,**C**) Population level. (**B**,**D**) Subject level, i.e., including random effects. (**A**,**B**) Linear axes scales. (**C**,**D**) Logarithmic axes scales. Dotted black lines, lines of identity. Broken blue lines, linear regressions.

The correlations between *C*(*t*) predicted with the final model and observed *C*(*t*) on both population and subject (including random effects) levels are shown in Figure 1. The remaining random effects of ln(*V*), ln(*k*), and *d_k_* of the final model are shown in Figures S17–S19. They were close to normally distributed as concluded from the histograms and QQnorm plots in Figure S22.

Considering that CL is the product of *V* and *k*, covariates for both ln(*V*) and ln(*k*) affect CL, except if canceled out, as in the case of *T*_inf_. Busulfan CL was thus dependent on TBW, *F*_mat_ as a maturation function of age, serum albumin, and underlying disease, namely ALL and HLH/XLP. Table S1 shows a summary of the fit PK parameters on population and subject levels.

**Figure 2:**
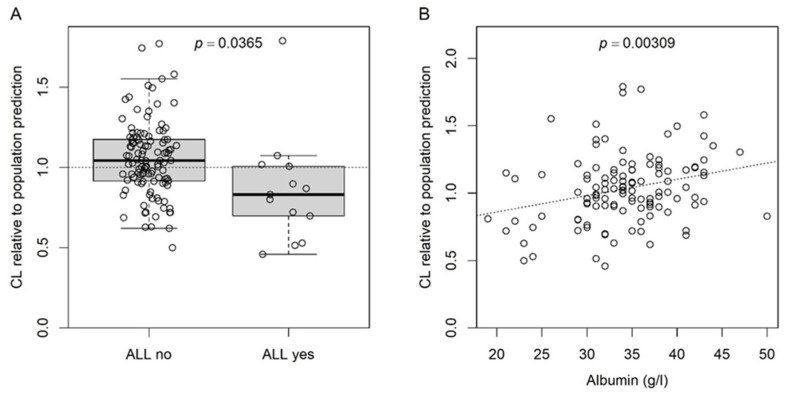
CL at the subject level relative to the respective predicted CL at the population level calculated before including the effects for ALL and albumin (on *k*) in the final model. (**A**) Boxplot for the effect of ALL; dotted line at 1, i.e., identity between predicted CL at subject and population level; *p*-value for Student’s *t*-test. (**B**) Scatter plot for the effect of serum albumin; dotted line, linear regression; *p*-value for the slope (slope = 0) of the linear regression.

To visualize the effects of ALL and albumin on busulfan CL, we recalculated the model without the two covariates. The respective CL of the individual subjects are shown in Figure 2 as multiples of the respective population predictions.

### 3.3. Model Validation

Each model was run at least 15 times with random start values (see Methods) to check for the reproducibility of the fit parameters. Applying the final model, typically all 15 runs converged with non-significant differences between the -2LL and robust fit parameters (Figure S23). Figures S24 and S25 show the distributions of the icwres and npde, respectively. The residues had a close to symmetrical distribution around 0, independent of the size of the predicted value or of time. This indicates that the structural model was adequately chosen. The tendency of a U-shape in the icwres plotted vs. observed *C*(*t*) justified the testing of a two-compartment model. However, -2LL was not significantly reduced with a two-compartment model compared to the final structural model 3** (Table 2). The histograms of the icwres and ndpe slightly deviated from a normal distribution (Figures S24 and S25). We investigated whether a proportional error model would bring the residues closer to a normal distribution. However, models 1 and 3** both revealed large *Ω*^2^ for ln(*V*) and an asymmetrical distribution of the residues and failed to calculate -2LL, indicating that the structural model 3** with an additive error model was adequate.

**Figure 3:**
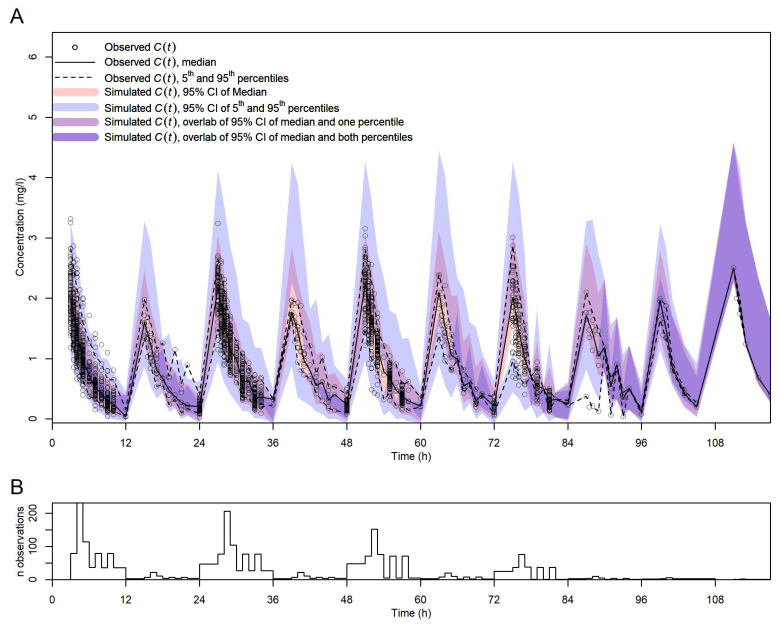
Visual predictive check for the final model. (**A**) vpc with observed and simulated *C*(*t*) vs. *t*. Symbols, lines, and shadings are as in the legend. (**B**) Number of observed *C*(*t*) per time point (no binning of time points).

Figure 3 shows the vpc plot. At most time points, the 5th and 95th percentiles of the observed data were within the respective percentiles’ 95% confidence intervals of the data simulated with the final model, indicating that the model was adequately built.

We further validated the model by bootstrapping. All fits with the 500 resampled datasets converged. The distributions of the fit parameter sets are shown in Figure S26 in comparison with the fit values and their confidence intervals (95%) from the original dataset (Table 3). The distributions of the fit parameters from resampling were in agreement with the fit parameters and confidence intervals revealed with the original dataset. The individual fit parameters from bootstrapping were normally distributed, as judged in Figure S26. The confidence intervals (95%) of the fit parameters from bootstrapping are shown in Table 3. None of these intervals included 0, confirming the significance of the fit parameters of the final model.

We finally simulated a situation in which the data would have been randomly split into a training and a test set at a ratio of 75/25. In the model building, BSA performed slightly better than TBW as a covariate for both ln(*V*) and ln(*k*). As BSA and TBW strongly correlate (Figure S16), we did not further take these discrepancies into account. In the training set, the effect of ALL on ln(*k*) did not significantly reduce -2LL; the reduction was −2.27 (*p* = 0.13). Apart from this, the model building with the training set identified the same covariates as the final model with the complete dataset. The observed vs. predicted *C*(*t*) of the training and test set and the fit parameters of the training set are shown in Figure S27.

### 3.4. Inter-Individual and Inter-Occasion Variability of Busulfan CL

**Figure 4:**
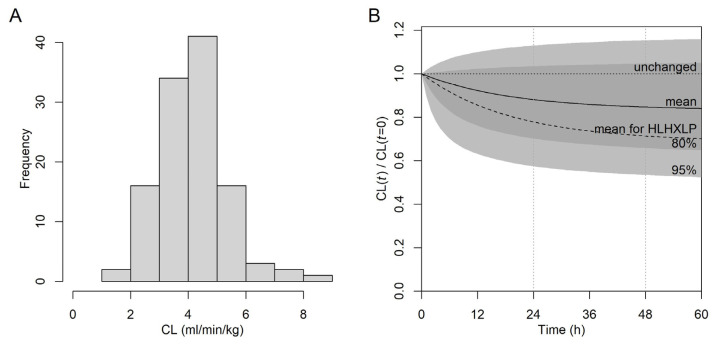
Inter- and intra-individual variability of busulfan CL based on the final model (Table 3) at the subject level. (**A**) Distribution of CL normalized to *W* in mL/min/kg at the start of therapy. (**B**) Simulated CL(*t*) normalized to the start CL (CL(*t* = 0)). Light gray area, predicted CL(*t*) within the 95% confidence interval; dark gray area, within the 80% confidence interval; solid line (“mean”), simulation from fixed effects (*d_k_*, ln(*κ_k_*)); broken line, simulation from fixed effects for diagnosis group HLH/XLP; horizontal dotted line, unchanged CL over *t*; vertical dotted lines at 24 h and 48 h.

The distribution of CL, as calculated from the fit *V* and *k* of the final model and normalized to *W* in mL/min/kg is shown in Figure 4. The significant effect of *d_k_* on *k* in the population PK model was in agreement with the reduction in non-parametric *k* with *t* (Figure S11). To test whether in addition to *k* also *V* was time-dependent, we expanded the final model in Table 3 by the parameters *d_V_* and *κ_V_* in analogy to *d_k_* and *κ_k_*. To avoid overparameterization, *κ_V_* was kept constant at the fit value of *κ_k_* in Table 3. The fit *d_V_* was 0.073, indicating that *V*, in contrast to *k* (*d_k_* changed from −0.167 to −0.273), did not substantially change over time (-2LL increased). As CL = *k* × *V*, the time dependence of *k* is directly translated to CL. The fit *d_k_* value of −0.167 with an SD of the random effects of 0.180 (Table 3) indicates an average reduction in *k* and CL by 16.7 ± 18.0% at a steady state. The fit *κ_k_* of exp(−2.965) indicates a half-life of ~13.4 h (ln(2)/exp(−2.965)) for the change in *k* and CL with *t*. This indicates that CL reaches constant values after 2–3 days of therapy. The simulated change in CL with *t* is shown in Figure 4.

### 3.5. Dose Adjustments and Busulfan Exposure

**Figure 5:**
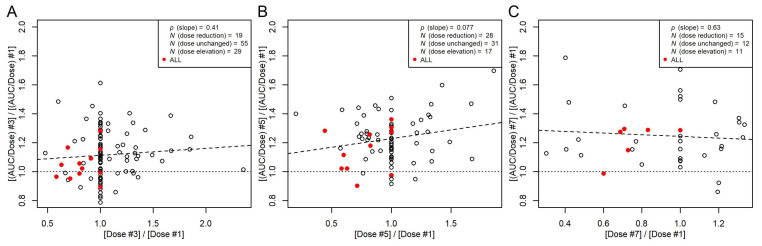
Ratios between non-parametric AUC/dose from the third (**A**), fifth (**B**), or seventh (**C**) dosing interval and AUC/dose at the first dosing interval plotted against the respective dose ratios. Filled red symbols, patients with ALL. Broken line, linear regression between the AUC/dose ratios and dose ratios with a *p*-value for the slope, indicating that the slope is not significantly different from 0. Dotted line, ratio of 1. Data with AUC measurements of even dose numbers are not included (*N* = 11). This includes one patient with ALL without dose adjustment.

Dose adjustments were required for several patients in our study in order to reach the target cAUC. These dose adjustments were, in general, realized for the second and subsequent doses based on the AUC of the first dosing interval with *C*(*t*) measurements. The data of these patients allowed us to assess the effect of the dose on busulfan PK at minimal influence of potentially confounding effects. As shown in Figure 5, the AUC normalized to dose (AUC/dose) was hardly affected by the dose.

Figure 5 shows that eight of the thirteen patients with ALL required a dose reduction, while none of them required a dose elevation. This agrees with the reducing effect of ALL on *k* (and CL) in the final model (Table 3), resulting in a higher AUC than expected.

## 4. Discussion

We retrospectively analyzed PK and clinical data in a primarily pediatric population undergoing busulfan-based conditioning for HSCT. To the best of our knowledge, this is the largest single-center study with strict twice-daily busulfan application in pediatric patients and serial PK measurements. Twice-daily administration of intravenous busulfan offers the advantage of higher flexibility in dose adjustment to reach the target cAUC. The high number of repeated measurements allowed for a detailed evaluation of endogenous and exogenous factors with respect to their effects on busulfan PK.

**Figure 6:**
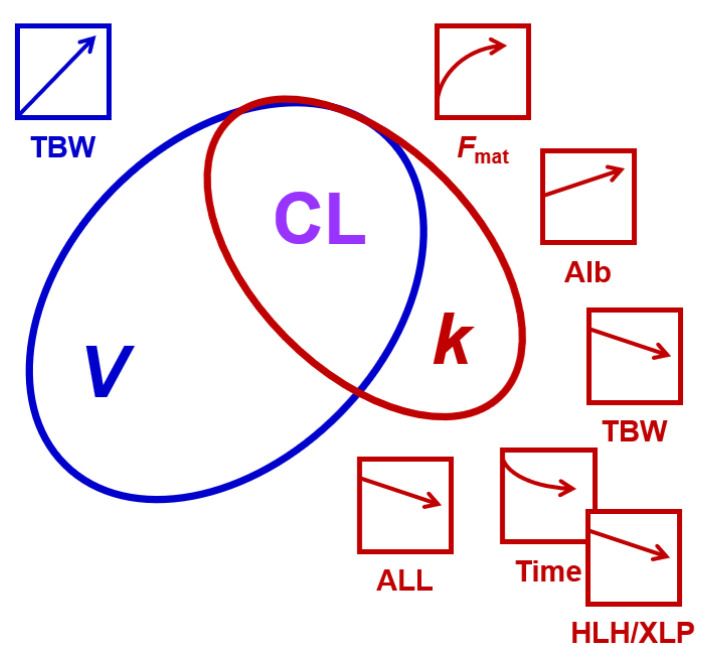
A schematic summary of the findings of this study. CL equals the product of *V* and *k*. *V* had a higher variability in the study population than *k*, as indicated by the size of the ellipses. The effects of patient characteristics on *V* and *k*, and thus CL, are indicated by the arrows and the respective color (blue, influencing *V*; red, influencing *k*). HLH/XLP amplifies the time-dependent reduction in *k* (indicated by the overlap of the respective symbols). Alb, albumin; ALL, acute lymphoblastic leukemia; *F*_mat_, maturation function of busulfan elimination process(es); HLH/XLP, hemophagocytic lymphohistiocytosis/X-linked lymphoproliferative disease; TBW, total body water. The artefactual effect of infusion time is not included.

To identify parameters explaining the inter-patient and inter-dose variability of busulfan PK in this pediatric cohort, we employed a non-linear mixed effects model. The relatively large dataset allowed us to search for covariates without prior inclusion of fixed allometric scaling exponents. Figure 6 provides a schematic summary of our findings. Not surprisingly, our data confirmed the dependency of busulfan CL on body size metrics [18,25,30,52]. This dependency resulted mainly from the correlation and near proportionality between *V* of busulfan and the TBW, calculated from *W*, *H*, age, and sex. The near agreement between *V* and TBW in our model suggests that busulfan is mainly distributed in the body water, as suggested in a previous analysis [53]. In our study, BSA performed similar to TBW as a covariate for *V*. Considering the near agreement between *V* and TBW and its (potential) physiological meaning (namely that busulfan distributes in total body water), we preferred keeping TBW as a covariate rather than the more commonly used parameter BSA or *W*. In addition to the effect of TBW on *V*, we identified several covariates for *k,* as summarized in Figure 6. Most previous population PK studies used CL and *V* as fit parameters [30], while we based our model on the elimination constant *k* and *V*. As CL is the product of *V* and *k*, covariates for both parameters also affect CL. Takahashi et al. recently published a comprehensive review on busulfan population PK studies [30]. Covariates for busulfan CL included *W*, BSA, FFM, normal-fat mass, ideal body weight, age, GSTA1 variants, and co-medication with fludarabine or fludarabine together with clofarabine, aspartate transaminase, and the time after first busulfan administration [30].

Including a function for the age-dependent maturation of *k* improved our model. The reduction in *k* and thus CL in patients < 1 year of age is in agreement with previous findings [18,23,24,25,54] and implies a maturation of the elimination mechanisms [55], which needs to be taken into account. Several approaches have been published to describe the maturation of busulfan CL [18,25,52]. The function introduced by McCune et al. [23] is based on postmenstrual age. As this simple one-parameter function significantly improved our model and as the residual random effects did not suggest any further common influencing factor, we did not test alternative maturation functions in our study.

A contribution of disease and intensity of the associated pre-conditioning treatment to the metabolism of busulfan was previously suggested [26,56]. In our cohort, ALL significantly reduced *k* without affecting *V* (resulting in a reduction in CL). The effect on *k* indicates a reduction in busulfan metabolism in these patients. The overall decrease in *k* and CL was on average 20% compared to patients without ALL. In agreement with this finding, dose reductions were frequently necessary in patients with ALL, while the diagnosis AML—another heavily pretreated group of patients—did not impact *k*. It remains speculative as to which factors contribute to the observed disease-associated difference in CL. As diagnosis and subsequent therapy of genetic diseases in children show age-related peaks, the impact of age and *W* on the CL of each disease group needs to be considered. Including TBW as the body size metric and the age-dependent maturation function as covariates in our model, we adjusted for these potential confounders.

Based on frequently performed PK measurements, which often exceeded the second day of busulfan application, we were able to profoundly analyze the inter-occasional variability of busulfan CL. By replacing the elimination rate constant *k* in the population PK model with a dynamic parameter, we were able to account for a change in *k* and CL with time. In our model, we used an exponential function to describe the change in *k* with time, while other studies used a step function [23,24], e.g., from the first to subsequent days, or a saturation function [54]. The half-life of the change in *k* in our analysis was 13.4 h, resulting in a constant CL 2–3 days after the start of busulfan therapy, which is in agreement with the reduction after the first day described in other studies [23,24]. The average reduction in CL was 16.7% in total. The corresponding estimated average reduction 24 h after therapy started is 12%. This result from population PK modeling was confirmed by the analysis of the individual patients’ datasets with a reduction in non-parametric *k* and CL from the first day to subsequent days. Here, we demonstrate that the CL of consecutive busulfan doses is significantly decreasing over time. However, CL and *k* were not reduced in all patients over time, as concluded from the variance of the random effects for *d_k_* and the comparison of the individual non-parametric *k* or CL. Our finding that CL was reduced in most (but not all) patients after one day of treatment is consistent with other large PK studies in pediatric patients, which demonstrated that CL on the first day was 8% to 15% higher [18,23,24,52,54,57] than subsequent days. This contrasts with PK studies in adult patients, where usually minimal inter-occasion variability of busulfan CL was observed [47,48,58,59]. As busulfan is metabolized by GSH conjugation, the depletion of intracellular GSH stores during therapy could, at the same time, explain both the overall decrease in CL and the variability in the change in CL over time [34,48,60]. A large fraction of our patients (69%) received at least two doses of NAC concomitantly with busulfan application with the intent to replenish GSH stores [37] and thus reduce (hepatic) side effects. We detected an effect of NAC on the observed *C*(*t*). Its effect in the PK model was slightly below our set limit for clinical relevance, albeit reaching significance. Its effect would correspond to a 10% increase in *V* and thus CL. In our study, NAC had no effect on the reduction in CL with time.

Patients with HLH/XLP as underlying conditions showed an even stronger decrease in busulfan CL (on average −31%) in follow-up doses, even though the initial CL did not differ from the remaining population. This may result from impaired liver function in these patients [61]. The capacity for replenishing GSH may be reduced, resulting in a stronger decrease in busulfan CL over time in these patients. Whatever the cause, the risk of overdosing and thus excessive toxicity of busulfan is considerably higher in HLH/XLP patients. Indeed, the observed incidence of SOS is increased in this population [15,62,63], and Marsit et al. reported a general association of significant decrease (>20%) in busulfan CL over multiple doses and subsequent occurrence of SOS [57].

As same-day therapeutic drug monitoring was performed during conditioning therapy, follow-up doses were adjusted based on the previously measured busulfan concentrations. Retrospectively, we analyzed the effect of the dose adjustment on busulfan PK. We expected a reduction in CL and a corresponding increase in AUC/dose with increasing the dose according to recently described saturation phenomena in GSH conjugation, either by saturating the involved GST or exceeding the capacity of replenishing GSH [34,48,60]. However, the observed effect was marginal, if any.

Furthermore, we analyzed a possible contribution of drug–drug interactions on the variability in busulfan CL, as patients undergoing conditioning for HSCT receive a substantial number of co-medications either as part of the conditioning regimen or adjuvant therapies. As discussed above, the depletion of GSH stores due to consumption in the course of busulfan metabolism was postulated as a cause for a reduction in busulfan clearance [24], and some of the administered co-medications are known to affect GSH levels, with paracetamol contributing to GSH depletion [36]. Our study showed no significant or clinically relevant effect of any of the administered co-medications on inter-patient, as well as inter-occasional variability of busulfan CL, as also reported in previous studies [57,64,65,66,67,68]. In particular, the administration of paracetamol prior to busulfan did not significantly affect busulfan CL in the PK model, irrespective of NAC administration. However, inspecting the observed vs. predicted *C*(*t*), we detected a slight underestimation of high *C*(*t*) if paracetamol was administered without NAC. While several reports disclosed a decrease in busulfan CL when combined with fludarabine [24,33,47,49,51], despite their proposed differential use of elimination routes [69], our findings corroborate studies suggesting no interaction [18,48,50].

In blood, almost 80% of busulfan is bound irreversibly to blood cells and plasma proteins, while the non-covalent binding of busulfan to plasma constituents is insignificant and unaffected by the busulfan concentration [31]. In our study, we observed a positive correlation between albumin levels and *k* (and thus CL). As albumin stoichiometrically and irreversibly binds to and thus inactivates busulfan, increased albumin levels could contribute to a more pronounced elimination of the drug [31,70]. Additionally, serum albumin is an indicator of liver function. Thus, lower albumin levels may suggest a disturbed hepatic function correlating with impaired busulfan metabolism. Both irreversible binding (resulting in loss of binding capacity) and an indication of liver dysfunction could explain the positive correlation between albumin levels and busulfan CL. However, albumin levels are influenced by a number of factors, including renal loss, inflammation, and nutritional status. A routine assessment of hepatic coagulation parameters (including antithrombin III)—likely a more sensitive indicator of acute liver dysfunction than albumin—in this patient cohort prior to conditioning did not reveal significant abnormalities.

Similarly to albumin, busulfan concentration in plasma is dependent on the hematocrit and potentially leukocyte concentration, as almost half of the active drug binds irreversibly to blood cells [31]. Our study showed a marginal change in busulfan PK with altered blood cell counts. While blood cell counts significantly improved our PK model in the forward inclusion phase of model building, they were excluded again in the backward elimination phase due to lack of significance.

No pharmacogenetic information was available in our retrospective study. Ben Hassine et al. [24] found a 12% reduction in CL for GSTA1 poor metabolizers and a 10% increase in CL for GSTA1 rapid metabolizers. Both groups each accounted for ~18% of the studied population. For comparison, the effect of the GSTA1 phenotype is of similar size as the effect we found for ALL. Including information on GSTA1 activity in our model might explain some, but not all, of the variability, as substantial unexplained variability remained in the study by Hassine et al. [24].

To conclude, we applied non-linear mixed effects modeling to evaluate the effects of patients’ clinical and laboratory parameters, as well as treatment-related factors, on busulfan PK in a pediatric cohort receiving strict twice-daily intravenous administration of busulfan. In addition to previously identified factors, plasma albumin level had a significant effect on busulfan CL and *t*_1/2_. Underlying disease emerged as a contributor to inter-patient and inter-occasional variability in CL and *t*_1/2_. In comparison to the initial dose, the majority of children demonstrated a substantial reduction in busulfan CL in subsequent dosing intervals. It remains speculative as to which factors contribute to the observed decrease in busulfan CL over time. Concomitant medication did only marginally impact busulfan CL or its inter-dose variability but was not significant in the final model. Our model was able to describe the busulfan PK of most patients in our study and may be useful in defining the initial dosing scheme more precisely. However, the remaining unexplained variability remained too high for a model-only approach for the prediction of cumulative busulfan doses. Consequently, the repeated TDM of busulfan with subsequent dose adjustment remains critical to achieve the desired disease-specific exposure in pediatric patients undergoing conditioning for autologous or allogeneic HSCT.

## Figures and Tables

**Table 1:** Patient characteristics.

Patients (*n* = 124)	Demographics
2015 (2010–2020)	Year of transplantation
4.3 (0.2–27.0)	Age (years)
23 (18%)	Age <1 (years)
95 (77%)	Age 1–18 (years)
6 (5%)	Age >18 (years)
17.2 (4.3–85.0)	Body weight (kg)
0.71 (0.25–2.06)	Body surface area (m^2^)
	Sex
89 (72%)	Male
35 (28%)	Female
	Indication
42 (34%)	Malignant disease
13 (10%)	ALL
12 (10%)	AML
6 (5%)	Neuroblastoma
11 (9%)	Others
4 (3%)	JMML
5 (4%)	MDS
1 (1%)	Ewing sarcoma
1 (1%)	Lymphoma
82 (66%)	Non-malignant disease
32 (26%)	CGD
14 (11%)	HLH or XLP
12 (10%)	Hemoglobinopathies
6 (5%)	β-Thalassemia major
6 (5%)	Sickle cell anemia
14 (11%)	Primary immunodeficiencies
6 (5%)	SCID
3 (2%)	Wiskott–Aldrich syndrome
5 (4%)	Others ^1^
8 (6%)	Metabolic diseases
4 (3%)	MPS
2 (2%)	X-ALD
1 (1%)	Other leukodystrophy
1 (1%)	Alpha-mannosidosis
2 (2%)	Thrombocytopenia
	Conditioning
82 (66%)	Bu/Flu
13 (10%)	Bu/Flu/TTP
4 (3%)	Bu/Flu/Mel
1 (1%)	Bu/Flu/CP
10 (8%)	Bu/Clo
7 (6%)	Bu/Mel
6 (5%)	Bu/CP/Mel
1 (1%)	Bu/CP
	Serotherapy
57 (46%)	Alemtuzumab
54 (44%)	ATG
13 (10%)	No
	Stem cell source
78 (63%)	Bone marrow
36 (29%)	Peripheral blood stem cell
10 (8%)	Umbilical cord blood
	HLA compatibility
50 (40%)	MUD
38 (31%)	MMUD
24 (19%)	MRD
5 (4%)	Haplo
7 (6%)	Auto
	Laboratory parameters
0.3 (0.17–0.41)	Hematocrit (L/L)
4.0 (0.16–20.9)	White blood cells (G/L)
61 (34–84)	Serum total protein (g/L)
34 (19–50)	Serum albumin (g/L)

^1^ Others: immunodeficiency DD WHIM syndrome, GATA-2 deficiency, IL-10 receptor-beta deficiency, hypereosinophilic syndrome, hyper-IgM syndrome. ALL, acute lymphatic leukemia; AML, acute myeloid leukemia; ATG, anti-thymocyte globulin; Auto, autologous; Bu, busulfan; CGD, chronic granulomatous disease; Clo, clofarabine; CP, cyclophosphamide; Flu, fludarabine; Haplo, haploidentical; HLH, hemophagocytic lymphohistiocytosis; JMML, juvenile myelomonocytic leukemia; MDS, myelodysplastic syndrome; Mel, melphalan; MMUD, mismatched unrelated donor; MPS, mucopolysaccharidosis; MRD, matched related donor; MUD, matched unrelated donor; SCID, severe combined immunodeficiency; TTP, thiotepa; X-ALD, X-linked adrenoleukodystrophy; XLP, X-linked lymphoproliferative disease. Data are shown in median (range) or number (%).

**Table 2:** Stepwise model building.

Step 1: Building the Basic Structural Model ^(a)^								
**Model**	**Number of Compartments**	**PK Parameters**	**Random Effects for**	**df ^(b)^**	**Δ-2LL ^(c)^**	**Ref. Model ^(d)^**	**Comment**	**Parameters in** Table 3
1 *	1	ln(*V*), ln(*k*)	ln(*V*), ln(*k*)	4	0	-		
2	1	ln(*V*), ln(*k*), *d_k_*_,_ ln(*κ_k_*)	ln(*V*), ln(*k*), *d_k_*_,_ ln(*κ_k_*)	8	−2245	1 *	Excluded, Shrinkage for ln(*κ_k_*) >40%	
3 *	1	ln(*V*), ln(*k*), *d_k_*_,_ ln(*κ_k_*)	ln(*V*), ln(*k*), *d_k_*	7	−2128	1 *	Critical Δ-2LL for Δ3 parameters: −7.82(*p* = 0.05)	*θ_V_*_1_^,^ *θ_k_*_1_^,^ *θ_dk_*_1_^,^ *θκ_k_*, all *Ω*^2^
4	2	ln(*V*), ln(*k*), ln(*V*_1_), ln(*k*_1_)	ln(*V*), ln(*k*), ln(*V*_1_)	7	−163	1 *		
Step 2: Search for covariates by forward inclusion based on model 3 * Inclusion criteria: Δ-2LL ^(c)^ ≤−3.84 (*p* = 0.05) and effect (absolute value, |*θ*|) ≥ 0.1 (≥ 10% in linear scale)								
**Model**	**Step 2a: Testing alternative body size metrics as covariates for ln(*V*)**			**df**	**Δ-2LL**	**Ref. model**	**Reason for not including**	**Parameter in** Table 3
5	ln(*W*) on ln(*V*)			8	−294.2	3 *	Model 9 *	
6	ln(BSA) on ln(*V*)			8	−304.6	3 *	Model 9 *	
7	ln(*H*) on ln(*V*)			8	−284.5	3 *	Model 9 *	
8	ln(FFM) on ln(*V*)			8	−288.3	3 *	Model 9 *	
9 *	ln(TBW) on ln(*V*)			8	−305.4	3 *		*θ* *_V_*_2_
	**Step 2b: Including additional covariates**							
10	Model 9 * with *T*_inf_ on ln(*V*)			9	−31.7	9 *		*θ* *_V_*_3_
11	Model 10 with *T*_inf_ on ln(*k*)			10	−15.2	10		*θ* *_k_*_6_
12	Model 11 with ALL on ln(*k*)			11	−14.8	11		*θ* *_θk_*_5_
13	Model 12 with HLH/XLP on *d_k_*			12	−6.94	12		*θ* *_dk_*_2_
14	Model 13 with Alb on ln(*k*)			13	−6.09	13		*θ* *_k_*_4_
15	Model 14 with ln(TBW) on ln(*k*)			14	−6.54	14		*θ* *_k_*_2_
16 **	Model 15 with ln(*F*_mat_) on ln(*k*)			15	−11.4	15		*θ* *_k_*_3_
17	Model 16 ** with ln(Leu) on ln(*k*)			16	−5.42	16 **	Step 3	
18 *	Model 17 with ln(Hct) on ln(*V*)			16	−5.25	16 **	Step 3	
**Step 3: Evaluation of covariates by backward exclusion** Exclusion criterium: Δ-2LL ≥ −6.63 (*p* = 0.01)								
17	Model 18 *, excluding ln(Hct) on ln(*V*)			15	−5.25	18	Δ-2LL ≥ −6.63	
16 **	Model 17, excluding ln(Leu) on ln(*k*)			15	−5.42	18	Δ-2LL ≥ −6.63	
	Excluding individual covariates from model 16 **			14	≤−7.40	16 **		
**Step 4: Search for the effects of co-medication**								
19	NAC on ln(*k*)			16	−0.03	16 **	Δ-2LL > −3.84	
20	NAC on ln(*V*)			16	−112.8	16 **	|*θ*| < 0.1 (0.099) ^(f)^	
21	Clofarabine with NAC on ln(*k*)			16	−1.48	16 **	Δ-2LL > −3.84	
22	Clofarabine with NAC on ln(*V*)			16	+0.62	16 **	Δ-2LL > −3.84	
23	Paracetamol w/o NAC on ln(*k*)			16	−4.33	16 **	|*θ*| < 0.1 (0.02)	
24	Paracetamol w/o NAC on ln(*V*)			16	−18.5	16 **	|*θ*| < 0.1 (−0.05)	
25	Fludarabine on ln(*k*)			16	+0.47	16 **	Δ-2LL > −3.84	
26	Fludarabine on ln(*V*)			16	+1.57	16 **	Δ-2LL > −3.84	

^(a)^ *, Model selected in each step; **, final model. ^(b)^ df, degrees of freedom. ^(c)^ -2LL of test model minus -2LL of reference model. ^(d)^ Ref. model, model compared to. ^(f)^ Fit parameter (effect). Alb, albumin; ALL, acute lymphoblastic leukemia; BSA, body surface area; FFM, fat-free mass; *F*_mat_, maturation of busulfan elimination process(es); *H*, body height; HLH/XLP, hemophagocytic lymphohistiocytosis/X-linked lymphoproliferative disease; Hct, hematocrit; *k*, first-order elimination rate constant; Leu, leucocyte count; NAC, N-acetylcysteine; *T*_inf_, duration of infusion; TBW, total body water; *V*, distribution volume; *W*, body weight; *Ω*^2^, variance of the random effects of a particular fit parameter; *θ*, fit parameter (fixed effect, i.e., population level).

**Table 3:** Fit parameters of the final model.

Fixed Effects							
Parameter ^(a)^	Reference Value ^(b)^	Fit Value	SE ^(c)^	Range in Population ^(d)^	Untransformed Fit Value ^(e)^	*p* ^(f)^	CI (95%) from Bootstrapping ^(g)^
$ln\left(V\right)=\theta_{V1}+\theta_{V2}\times (\ln\left(TBW\right)-\ln\left(10L\right))+\theta_{V3}\times (T_{inf}-3h)\;V=e^{\theta_{V1}}\times {\left(TBW/\left(10L\right)\right)}^{\theta_{V2}}\times e^{\theta_{V3}\times \left(T_{inf}-3h\right)}$							
$\theta_{V1}$ *,* ln(*V*, L) at reference values		2.459	0.0189		11.70 L	-	2.432, 2.491
$\theta_{V2}$ , effect of Δln(TBW, L)	10 L	0.931	0.0233	−1.21; 1.40	-	<0.001	0.872, 0.987
$\theta_{V3}$ , effect of Δ*T*_inf_ (h)	3 h	0.226	0.0356	0; 0.23	1.254	<0.001	0.134, 0.329
$ln\left(k\right)=\theta_{k1}+\theta_{k2}\times \left(ln\left(TBW\right)-ln\left(10L\right)\right)+\theta_{k3}\times ln\left(F_{mat}\right)+\theta_{k4}\times \left(ln\left(Alb\right)-ln\left(30g/L\right)\right)+\theta_{k5}\times ALL+\theta_{k6}\times \left(T_{inf}-3h\right)\;k=e^{\theta_{k1}}\times {F_{mat}}^{\theta_{k2}}\times {\left(TBW/\left(10L\right)\right)}^{\theta_{k3}}\times {\left(Alb/\left(30g/L\right)\right)}^{\theta_{k4}}\times e^{\theta_{k5}\times ALL}\times e^{\theta_{k6}\times \left(T_{inf}-3h\right)}$							
$\theta_{k1}$ , ln(*k*, h^−1^) at reference values		−1.007	0.0329		0.365 h^−1^	-	−1.065, −0.952
$\theta_{k2}$ , effect of Δln(TBW, L)	10 L	−0.189	0.0411	−0.28; 0.24	-	< 0.001	−0.291, −0.106
$\theta_{k3}$ , effect of ln(*F*_mat_)	0	0.697	0.2001	−0.40; 0	-	< 0.001	0.348, 1.061
$\theta_{k4}$ , effect of Δln(Alb, g/L)	30 g/L	0.331	0.1073	−0.15; 0.16	-	0.001	0.090, 0.653
$\theta_{k5}$ , effect of ALL	No ALL	−0.210	0.0592	−0.21; 0	0.810	< 0.001	−0.398, −0.030
$\theta_{k6}$ , effect of Δ*T*_inf_ (h)	3 h	−0.161	0.0420	−0.16; 0	0.851	< 0.001	−0.264, −0.085
$k^{\prime }=k\times \left({-d}_{k}\times \left(exp\left({-}_{k}\times t\right)-1\right)+1\right)$ $;\;d_{k}=\theta_{dk1}+\theta_{dk2}\times HLH/XLP$ $;{\;}_{k}=e^{\theta_{k}}$							
$\theta_{dk1}$ , *d_k_* (h^−1^) at no HLH/XLP	-	−0.167	0.0191		−0.167	-	−0.198, −0.122
$\theta_{dk2}$ , effect of HLH/XLP	No HLH/XLP	−0.145	0.0540	−0.15; 0	−0.145	0.0035	−0.255, −0.030
$\theta_{k}$ , ln(*κ_k_*, h^−1^)	-	−2.965	0.1094		0.0516 h^−1^	-	−3.204, −2.390
**Inter-individual variability: variance** **Ω^2^ (standard deviation** **Ω) of the random effects**							
**Parameter**		**Fit** *Ω*^2^ (*Ω*)	**SE of** *Ω* ^2^	**Shrinkage**			
*Ω*^2^ (*Ω*) of ln(*V*)	-	0.029 (0.169)	0.0039	5.5%			
*Ω*^2^ (*Ω*) of ln(*k*)	-	0.033 (0.182)	0.0048	12.5%			
*Ω*^2^ (*Ω*) of *d_k_*	-	0.032 (0.180)	0.0047	12.5%			
**Residual error**							
Residual error with SE	-	0.076	0.0008			-	-

^(a)^ *k*, first-order elimination rate constant; *V*, distribution volume; *d_k_*, amplitude of a change in *k* with time; *κ_k_*, exponent of a change in *k* with time. Reading examples: $\theta_{V1}$ , ln(*V*, in L) at reference values for the covariates, i.e., TBW = 10 L and *T*_inf_ = 3 h. $\theta_{V2}$ , effect of Δln(TBW, in L), i.e., ln(TBW) − ln(10 L), on ln(*V*). $\theta_{V3}$ , effect of Δ*T*_inf_ (h), i.e., difference of *T*_inf_ to 3 h, which is 0 for *T*_inf_ = 3 h and 1 h for *T*_inf_ = 4 h, on ln(*V*). For more details, see equations in the gray table inserted above the respective parameters. *Ω*^2^ and *Ω*, variance and standard deviation of the random effects of the indicated fit parameters. ^(b)^ Reference values, rounded values close to the median of the population. ^(c)^ SE, standard error of the fit value. ^(d)^ Range in population, e.g., range of $\theta_{V2}\times \left(ln\left(TBW\right)-ln\left(10L\right)\right)$ ; ^(e)^ untransformed fit values where meaningful: exp(*θ*) if *θ* corresponds to a logarithmic (ln) parameter, otherwise directly *θ*. ^(f)^ *p* of the fit parameter. ^(g)^ See Section 3.3. Alb, albumin; ALL, acute lymphoblastic leukemia; *F*_mat_, maturation function of busulfan elimination process(es); HLH/XLP, hemophagocytic lymphohistiocytosis/X-linked lymphoproliferative disease; TBW, total body water; *T*_inf_, duration of infusion.

## Data Availability

Secure access to the data presented in this study is available upon request (M.M.H.-H.) according to the data-sharing policies of SPHN. The data are not publicly available due to confidentiality reasons (Swiss Human Research Act, 810.30).

## Supplementary Materials

The following supporting information can be downloaded at www.mdpi.com/xxx/s1, Figure S1: Distribution of parameters of the studied population; Figure S2: Conditioning regimens performed and concomitant medication given during treatment before HSCT; Figure S3: Distribution of the observed busulfan plasma concentrations. Figure S4: Non-compartmental AUC vs. patient characteristics. Figure S5: Non-parametric *C*_max_ vs. patient characteristics. Figure S6: Non-parametric CL vs. patient characteristics. Figure S7: Non-parametric *V* vs. patient characteristics. Figure S8: Non-parametric *t*_1/2_ vs. patient characteristics. Figure S9: Non-parametric MRT vs. patient characteristics. Figure S10: Correlations between non-parametric *V*, *k,* and CL. Figure S11: Comparison of non-parametric *k* of the 1^st^ dose to *k* of the following doses; Figure S12: Comparison of non-parametric CL of the 1^st^ dose to *k* of the following doses; Figure S13: Random effects of ln(*V*) plotted against potential covariates (before including covariates); Figure S14: Random effects of ln(*k*) plotted against potential covariates (before including covariates); Figure S15: Random effects of *d_k_* plotted against potential covariates (before including covariates); Figure S16: Relations between patient characteristics; Figure S17: The remaining random effects of ln(*V*) of the final model plotted against potential covariates; Figure S18: The remaining random effects of ln(*k*) of the final model plotted against potential covariates; Figure S19: The remaining random effects of *d_k_* of the final model plotted against potential covariates; Figure S20: Effects of co-medication on the observed busulfan plasma concentrations; Figure S21: Comparison of *d_k_* in the presence and absence of NAC; Figure S22: QQnorm plots and histograms of the distributions of the remaining random effects of the final model; Figure S23: Fit parameters of the final model from 15 runs plotted against the respective -2LL; Figure S24: Distribution of the icwres; Figure S25: Distribution of the npde. Figure S26: Results from bootstrapping; Figure S27: Observed vs. predicted *C*(*t*) after splitting the data into a training and a test set. Table S1: PK parameters for the final model and the studied patient population.
