# Supplementary material for: Population Pharmacokinetic Modeling for Twice-Daily Intravenous Busulfan in a Large Cohort of Pediatric Patients Undergoing Hematopoietic Stem Cell Transplantation—A 10-Year Single-Center Experience
Source: Pharmaceutics. 2023 Dec 20;16(1):0. doi: 10.3390/pharmaceutics16010013 (PMC11154452; doi:10.3390/pharmaceutics16010013)
Supplement: Supplementary file 1 [file pharmaceutics-16-00013-s001.zip › pharmaceutics-2707966-supplementary.pdf]

pharmaceutics

MDPI

Supplementary Material

Population pharmacokinetic modelling for twice-daily intravenous busulfan in a large cohort of pediatric patients undergoing hematopoietic stem cell transplantation – a 10-year single-center experience.

Katharina M. Schreib[1], Dominic S. Bräm[2], Ulrike Zeilhofer[1], Daniel Müller[3], Tayfun Güngör[1], Stefanie D. Krämer[2*] and Mathias M. Hauri-Hohl[1*]

1 Department of Stem Cell Transplantation, University Children's Hospital Zurich – Eleonore Foundation & Children`s Research Center (CRC), University of Zurich, Switzerland; katharina.schreib@kispi.uzh.ch (K.M.S.); ulrike.zeilhofer@kispi.uzh.ch (U.Z.); tayfun.guengoer@kispi.uzh.ch (T.G.); mathias.hauri@kispi.uzh.ch (M.M.H.-H.)

2 Institute of Pharmaceutical Sciences, Department of Chemistry and Applied Biosciences, ETH Zurich, Switzerland; dominic.braem@ukbb.ch (D.S.B.); stefanie.kraemer@pharma.ethz.ch (S.D.K.)

3 Institute for Clinical Chemistry, University Hospital Zurich, Switzerland; e-mail@e-mail.com

* These authors contributed equally.

* Correspondence: mathias.hauri@kispi.uzh.ch (M.H.-H.); stefanie.kraemer@pharma.ethz.ch (S.D.K.)

Table S1. PK parameters for the final model and for the studied patient population. Population level (subject level).

Maximal value	Median value	Mean value	Minimal value	
59.28 (86.27)	12.38 (12.54)	16.67 (16.93)	3.498 (3.061)	V (l)
1.001 (1.644)	0.7293 (0.74)	0.7545 (0.7622)	0.5373 (0.4118)	V (l/kg)
70.09 (115.1)	51.05 (51.8)	52.82 (53.35)	37.61 (28.82)	V (l per 70 kg)
256.4 (351.5)	74.95 (73.25)	86.33 (88.96)	18.58 (14.47)	CL (ml/min)
5.686 (8.466)	4.234 (4.175)	4.154 (4.277)	2.556 (1.766)	CL (ml/min/kg)
127.6 (184.4)	104.8 (106.3)	103 (106)	67.91 (47.38)	CL (ml/min/m^2)
398.0 (592.6)	296.4 (292.2)	290.8 (299.4)	178.9 (123.6)	CL (ml/min/70 kg)
220.7 (319.1)	181.3 (183.9)	178.2 (183.3)	117.5 (81.96)	CL (ml/min/1.73 m^2)
3.604 (4.512)	2.062 (2.032)	2.132 (2.151)	1.566 (1.204)	$t_{1/2}$ (h)
-0.167 (0.231)	-0.167 (-0.185)	-0.183 (-0.189)	-0.312 (-0.713)	d_k (-)
	13.44 (-)			$T_{1/2}$ (ln(2)/κ_k) (h)

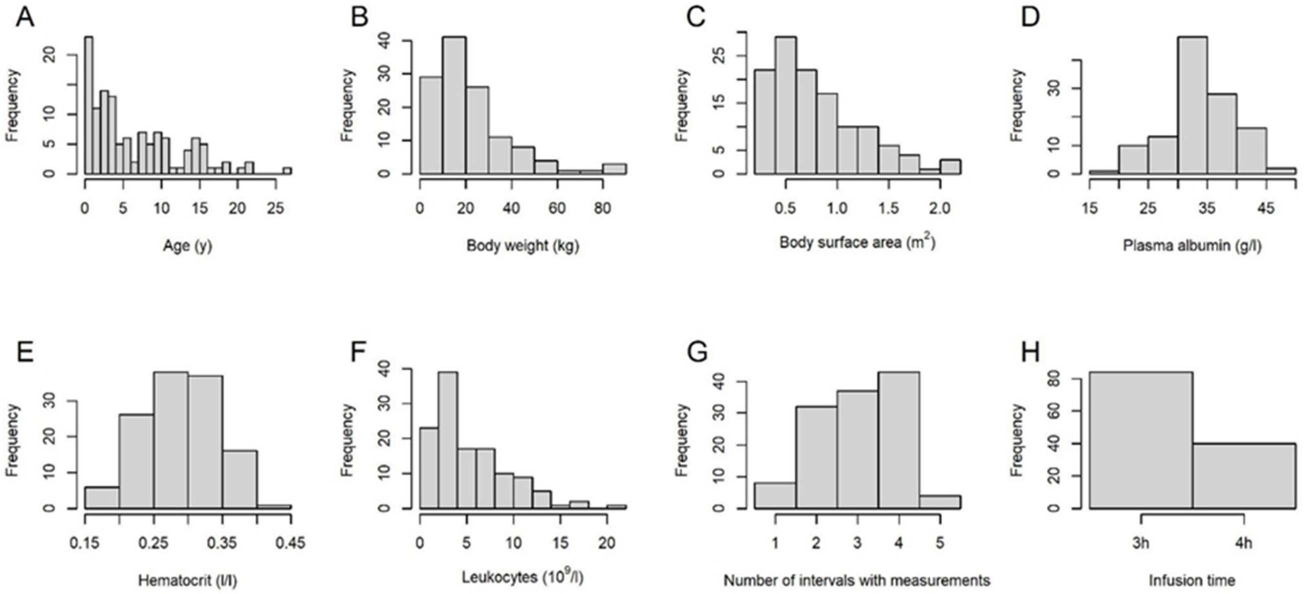

Figure S1. Distributions of age (**A**), body weight (**B**), body surface area (**C**), plasma albumin (**D**), hematocrit (**E**) leukocytes count (**F**), number of dosing intervals with *C*(*t*) measurements performed per patient (**G**) and infusion time (**H**) of the studied population. See Table 1 for other parameters.

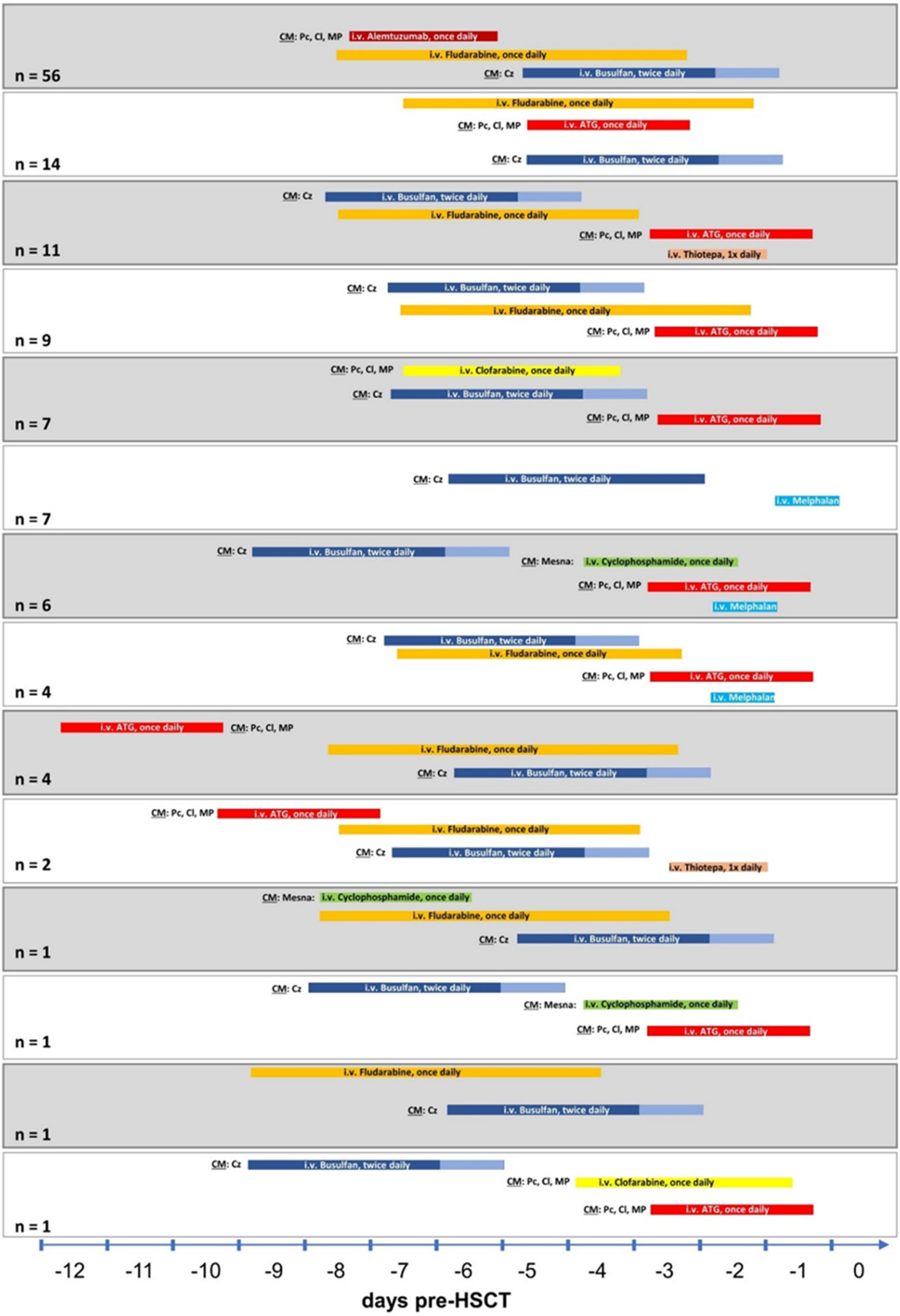

Figure S2. (previous page). Conditioning regimens performed and concomitant medication given during treatment before HSCT. n, numbers of patients with corresponding treatment regimen. Colored bars indicate the time frame of administered medication. Blue, busulfan; orange,

fludarabine; yellow, clofarabine; green, cyclophosphamide; bright red, ATG; dark red, alemtuzumab; salmon, thiotepa; turquoise, melphalan. The shade of blue depends on the number of busulfan doses required to reach cAUC. Abbreviations: ATG, anti-thymocyte globulin; CM, concomitant medication; Cl, clemastine; Cz, clonazepam; MP, methylprednisolone; Pc, paracetamol. NAC is not shown in the Figure; 69% of the patients received at least two doses NAC concomitantly with busulfan application.

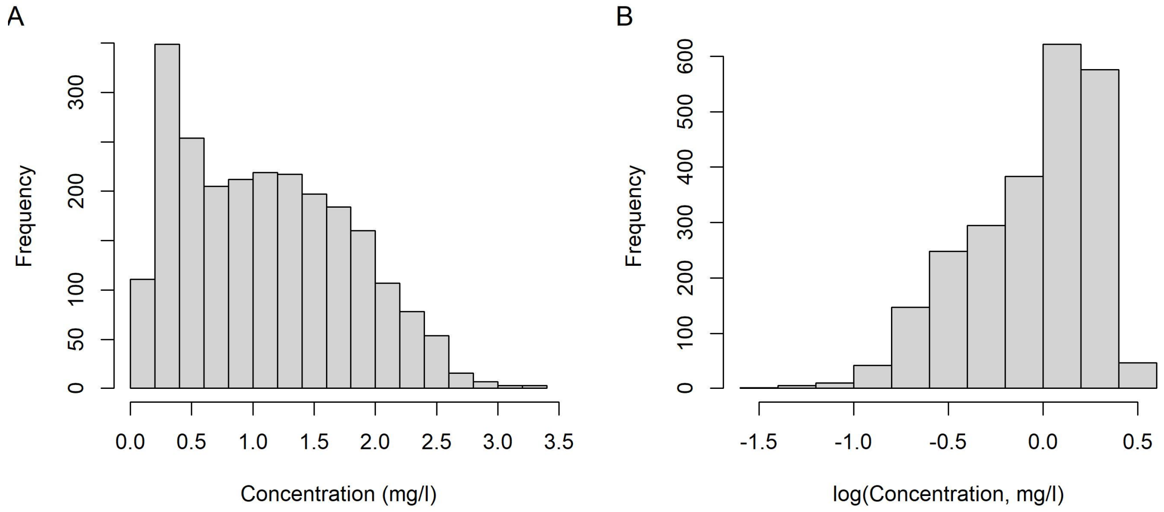

Figure S3. Distribution of the observed busulfan plasma concentrations. **A**) Linear concentration scale. **B**) Logarithmic (base 10) concentration scale.

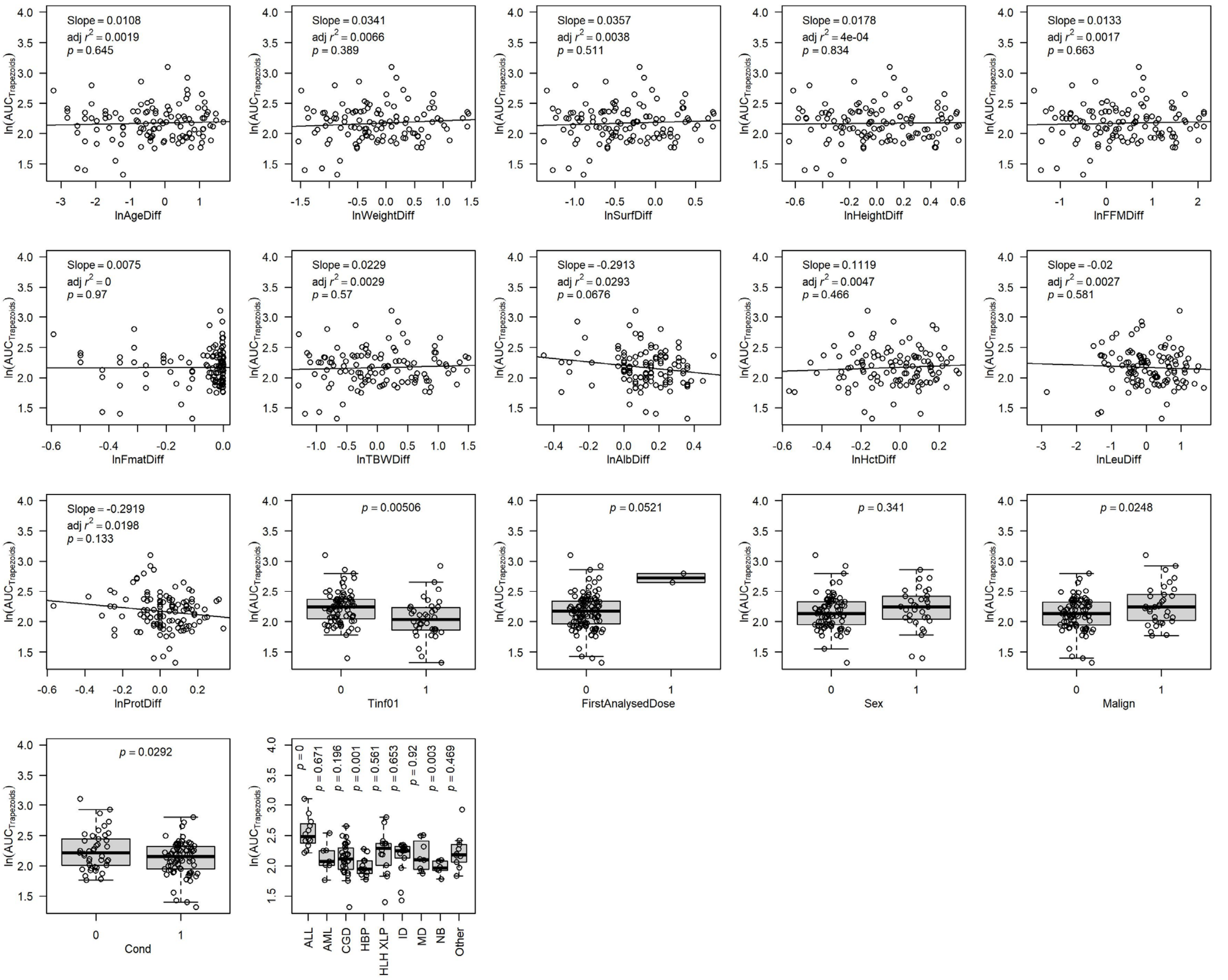

Figure S4. Non-parametric ln(AUC) of the first dosing interval, calculated from the trapezoids of the $C(t)$ *vs* t curves plotted *vs* patient characteristics. AUC in mg×h/l. Continuous patient characteristics are plotted as ln(value)-ln(reference value), where the reference value is close to the population mean (reference values are stated in Table 3 of the manuscript). Statistics in Figure inserts. For continuous patient characteristics, slope, adjusted r^2 and p for slope=0 of linear regressions (lines). For patient characteristics with two alternative states, p for two-sided, homoscedastic t-test. For patient characteristics with multiple alternatives, p for t-tests of one category compared to the rest, without post-hoc corrections for multiple comparisons.

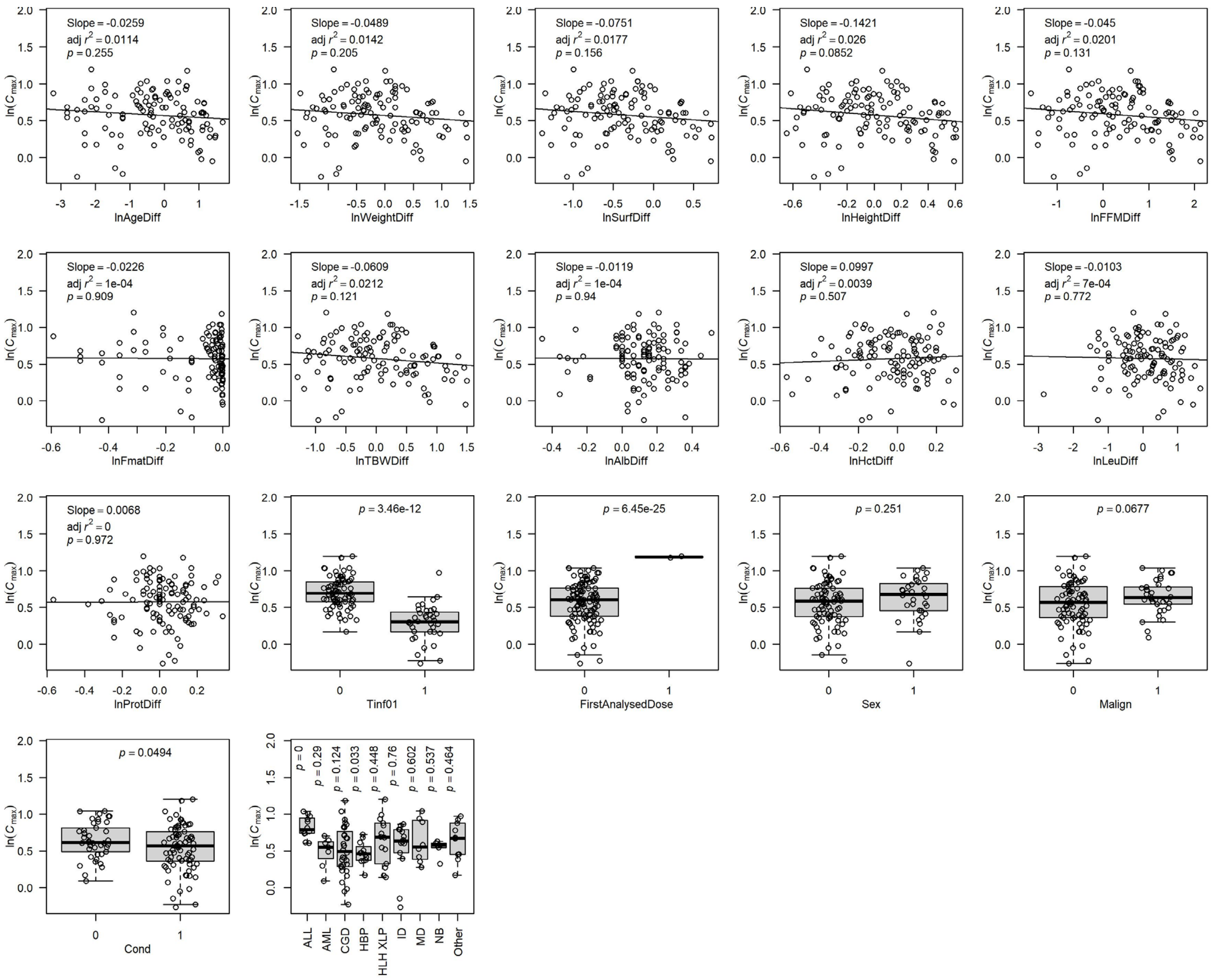

Figure S5. The maximal observed $C(t)$ of the first dosing interval, as $\ln(C_{max})$, plotted *vs* patient characteristics. C_{max} in mg/l. Further details, see Figure S4.

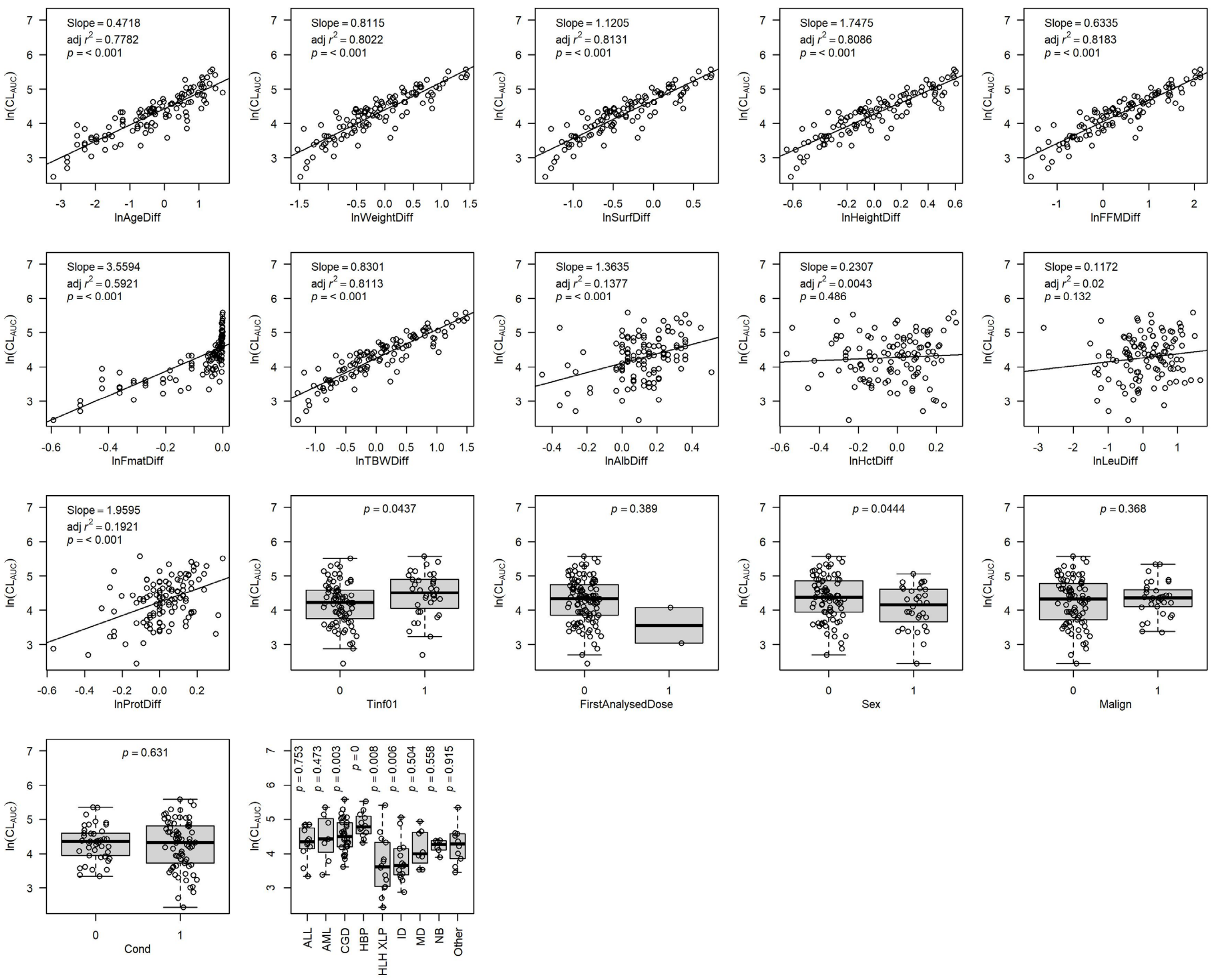

Figure S6. Non-parametric ln(CL) of the first dosing interval, with CL=dose/AUC, plotted *vs* patient characteristics. CL in ml/min. AUC determined from the trapezoids of the $C(t)$ *vs* t plot. Further details, see Figure S4.

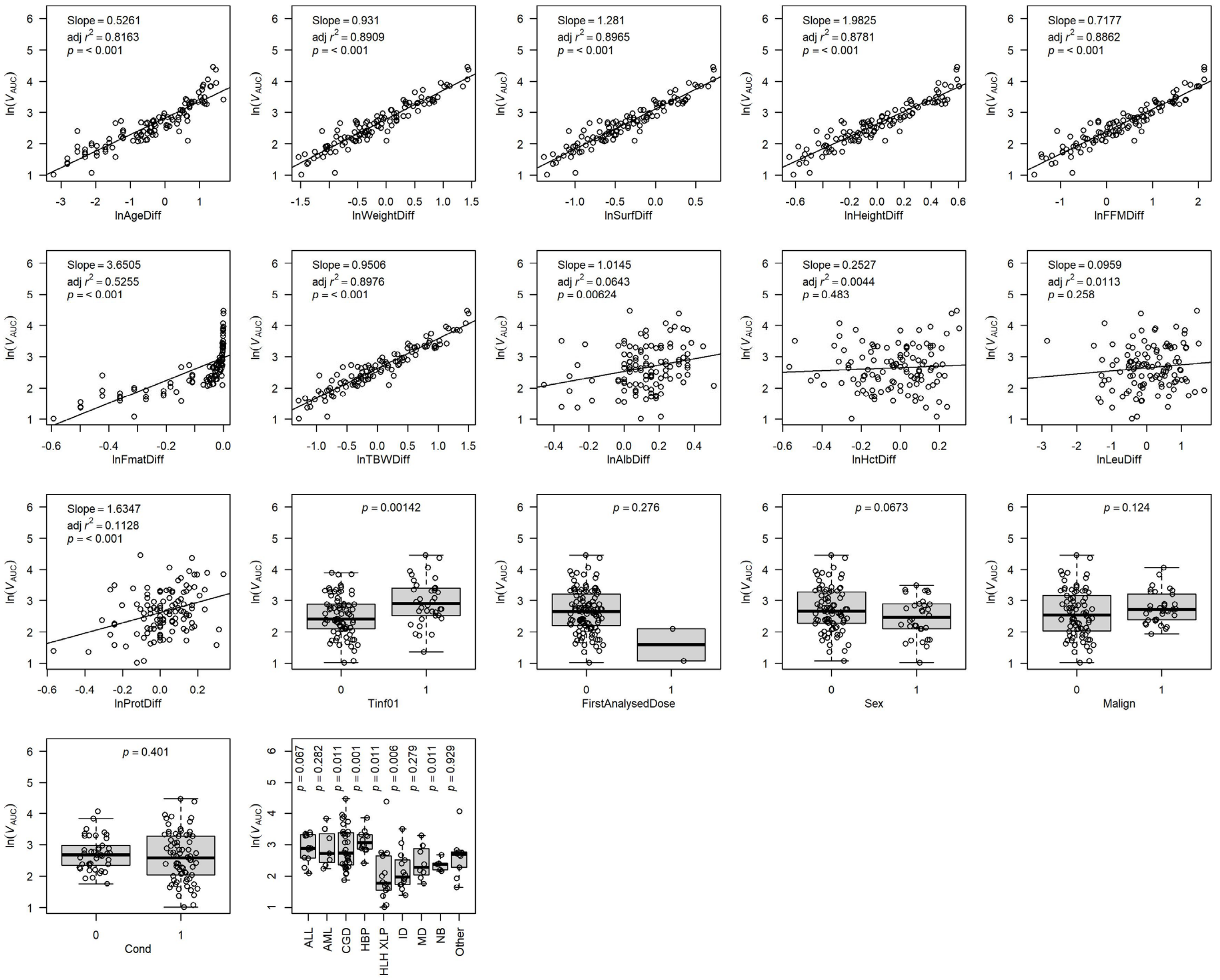

Figure S7. Non-parametric ln(V) of the first dosing interval, plotted *vs* patient characteristics, with V=dose/(AUC × k) with AUC calculated from the trapezoids of the $C(t)$ *vs* t plot and k corresponding to the slope of the decreasing phase of ln($C(t)$) *vs* t, both of the first dosing interval. V in liter. Further details, see Figure S4.

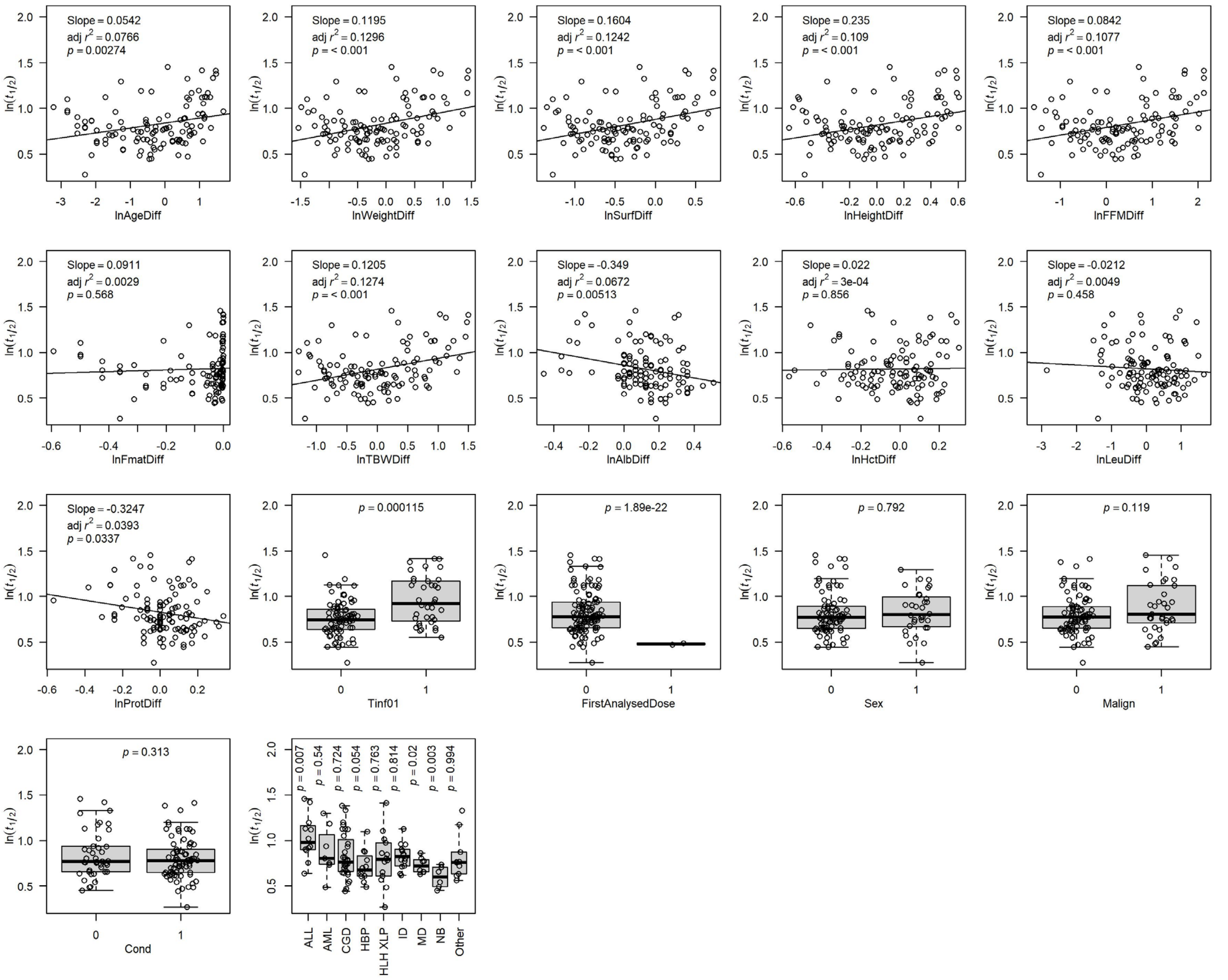

Figure S8. $\ln(t_{1/2})$ of the first dosing interval, plotted *vs* patient characteristics. $t_{1/2} = \ln(2)/k$, with k as the slope of the decreasing phase of $\ln(C(t))$ *vs* t of the first dosing interval. $t_{1/2}$ in h. Further details, see Figure S4.

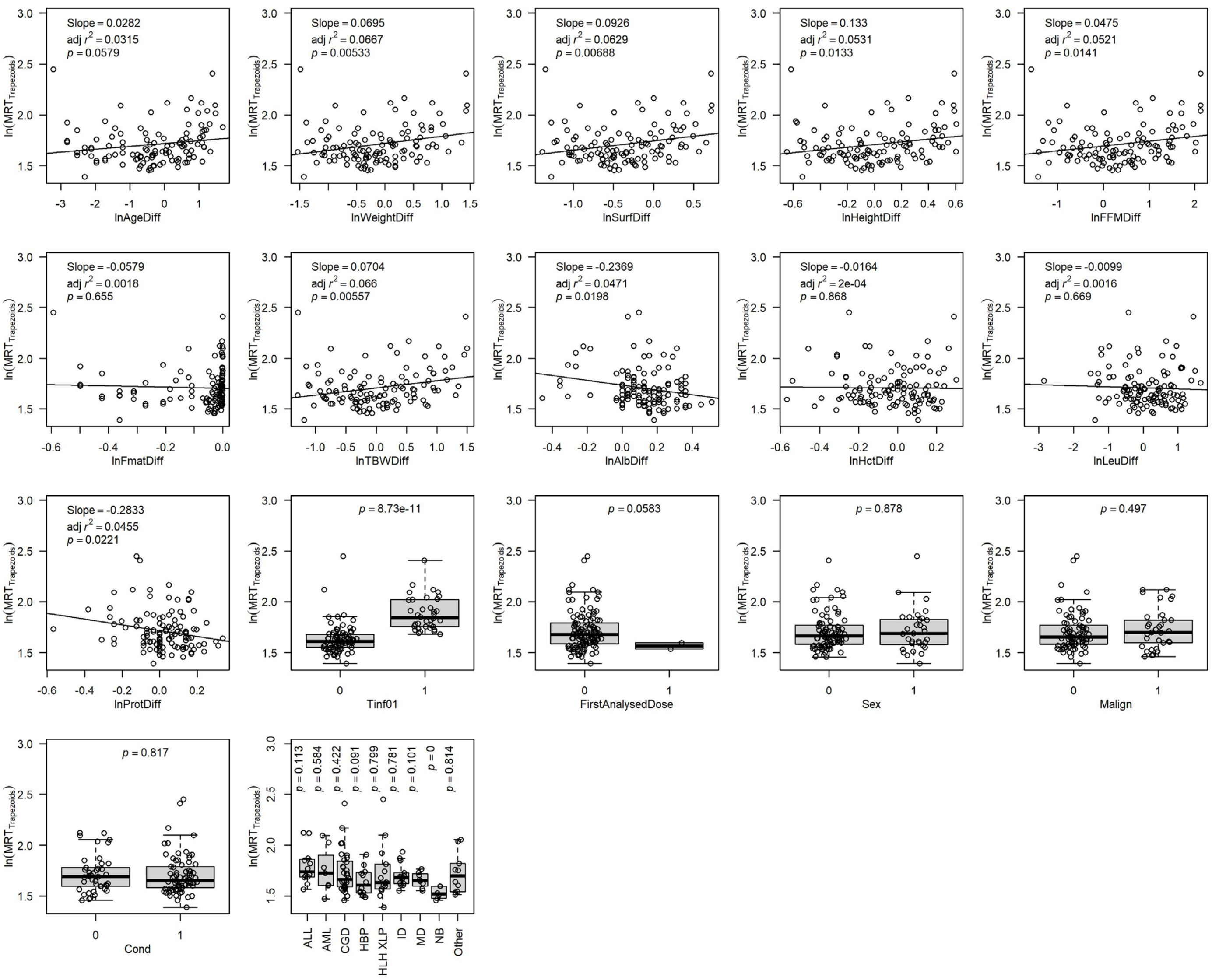

Figure S9. ln($MRT_{Trapezoids}$) plotted *vs* patient characteristics. MRT in h. Further details, see Figure S4.

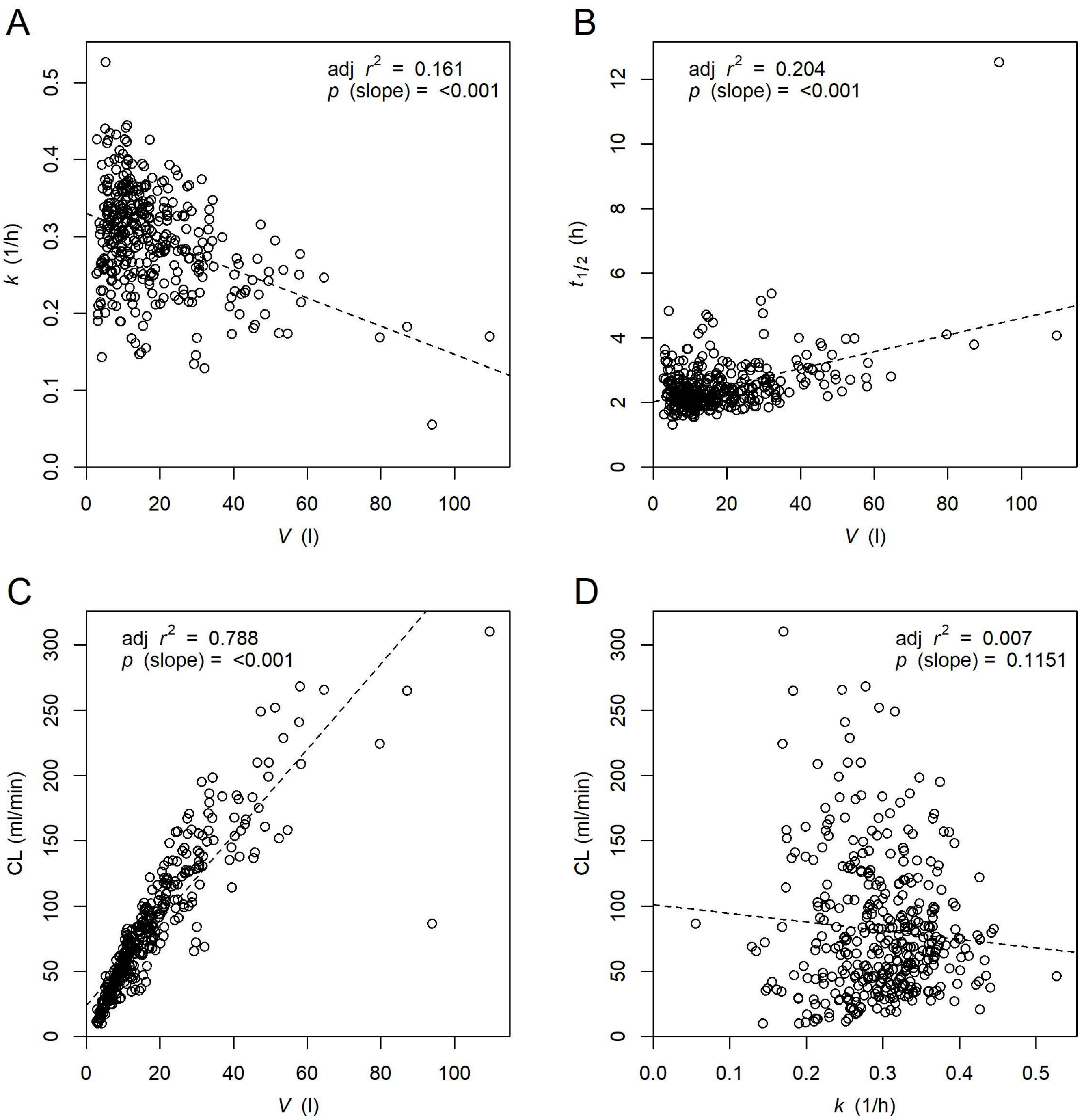

Figure S10. Relationship between non-parametric k, V, $t_{1/2}$ and CL. Broken lines, linear regressions, with adjusted r^2 and p (for slope=0) in the Figure panes. CL correlated stronger with V than with k.

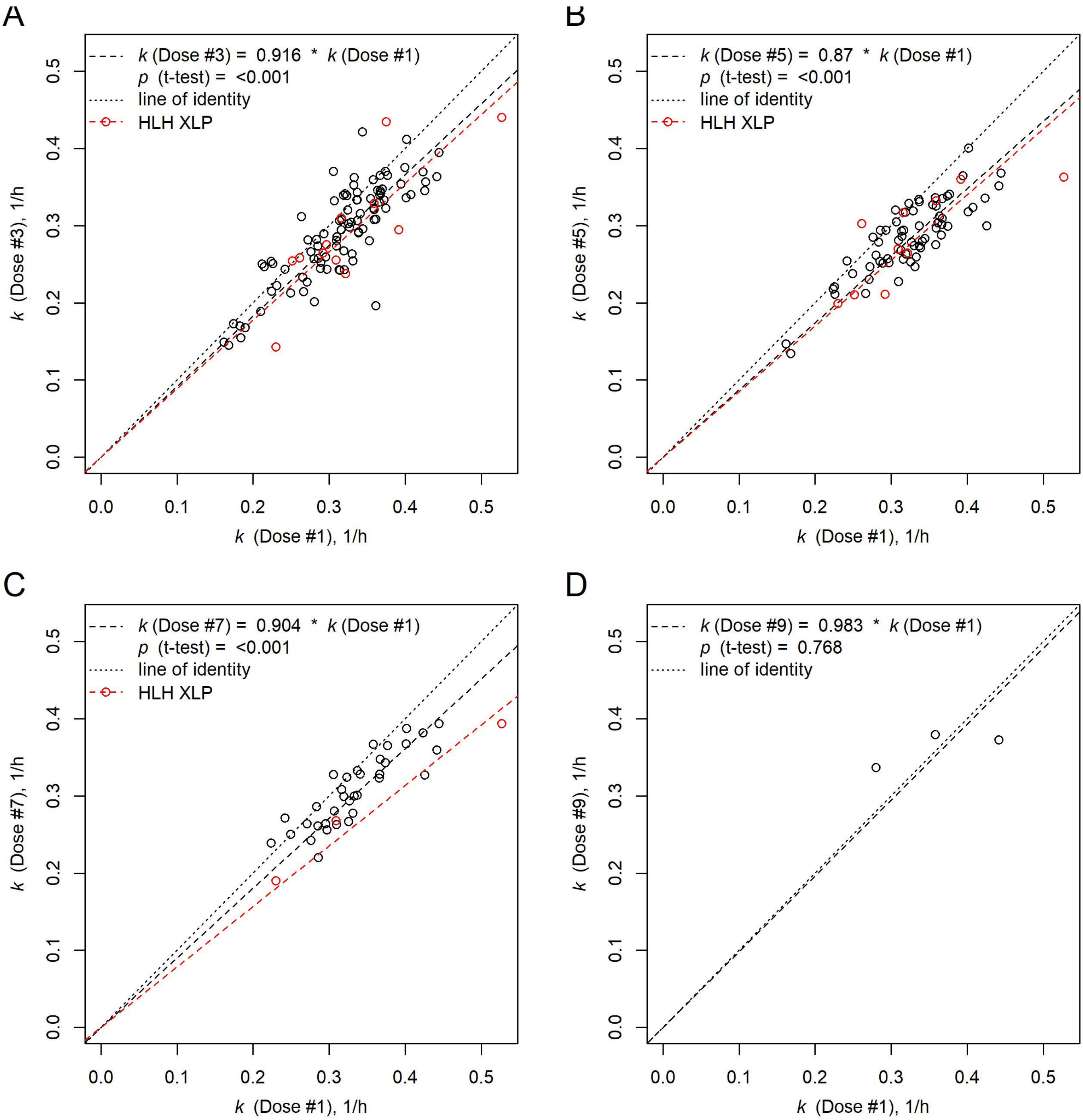

Figure S11. Comparison of k of the 1st dose to k of the following doses. k were determined by linear regression of decreasing phase of $\ln(C(t))$ *vs* t. Broken lines, linear regression between k of the indicated doses and k(Dose #1), with slopes as indicated; intercepts were forced to 0. Dotted lines, lines of identity for comparison. k of the 3rd (**A**), 5th (**B**) and 7th (**C**) doses were significantly lower than k(Dose #1) as concluded from the plots and the p-values comparing k of the indicated dosing interval to k of the 1st dosing interval (two-sided, homoscedastic). Note, only 3 measurements for the 9th dose (**D**). Red symbols and red broken lines, data of patients with HLH/XLP and linear regression lines through 0, respectively.

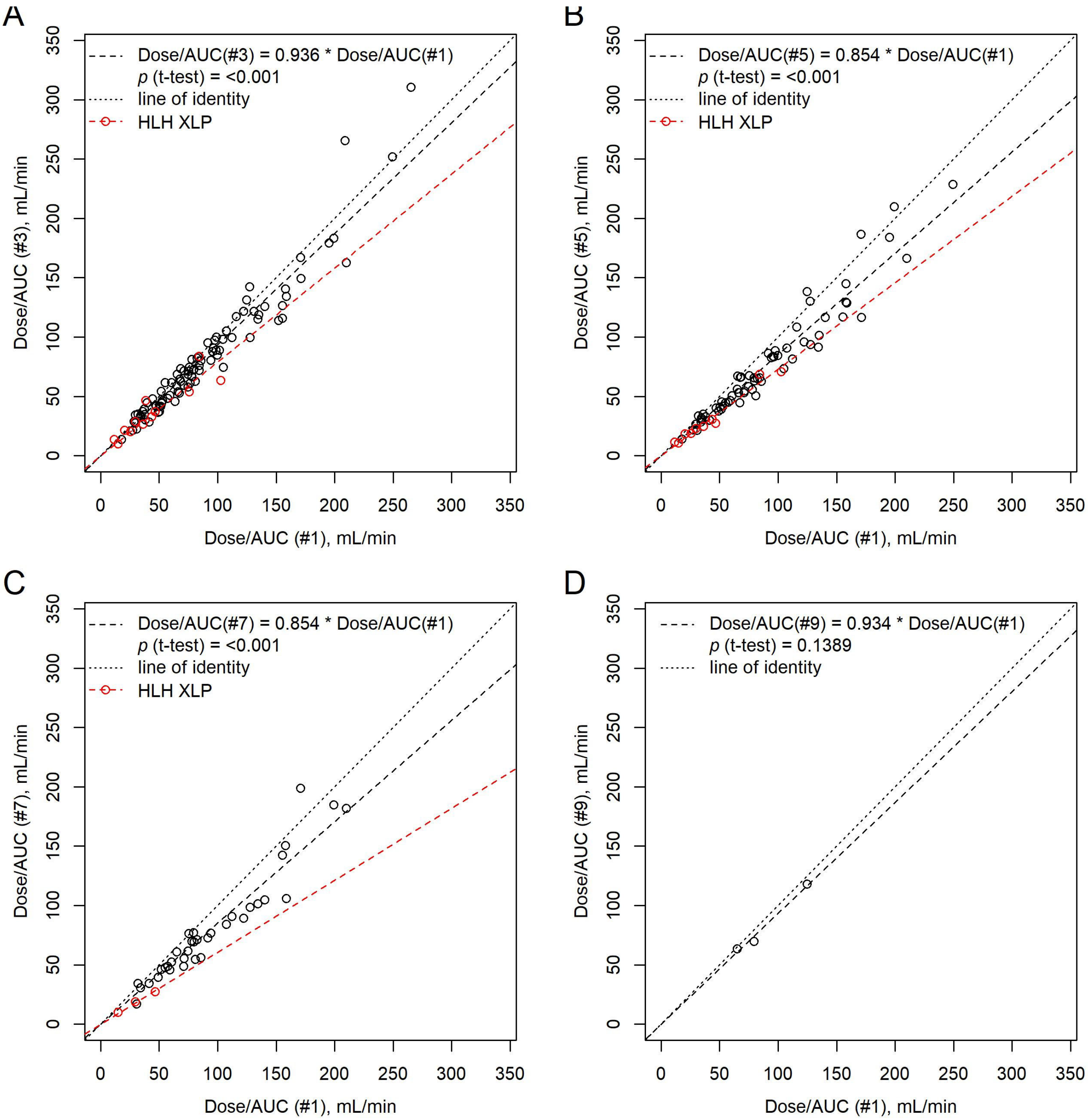

Figure S12. Non-parametric CL (dose/AUC) from the 3rd (**A**), 5th (**B**), 7th (**C**) and 9th (**D**) dose to the CL of the 1st dose. The AUC were calculated by the trapezoidal method. Broken lines, linear regressions (intercept forced to 0) with statistics as in Figure S11. Dotted lines, lines of unity. Red symbols and red broken lines, data of patients with HLH/XLP and linear regression lines through 0, respectively.

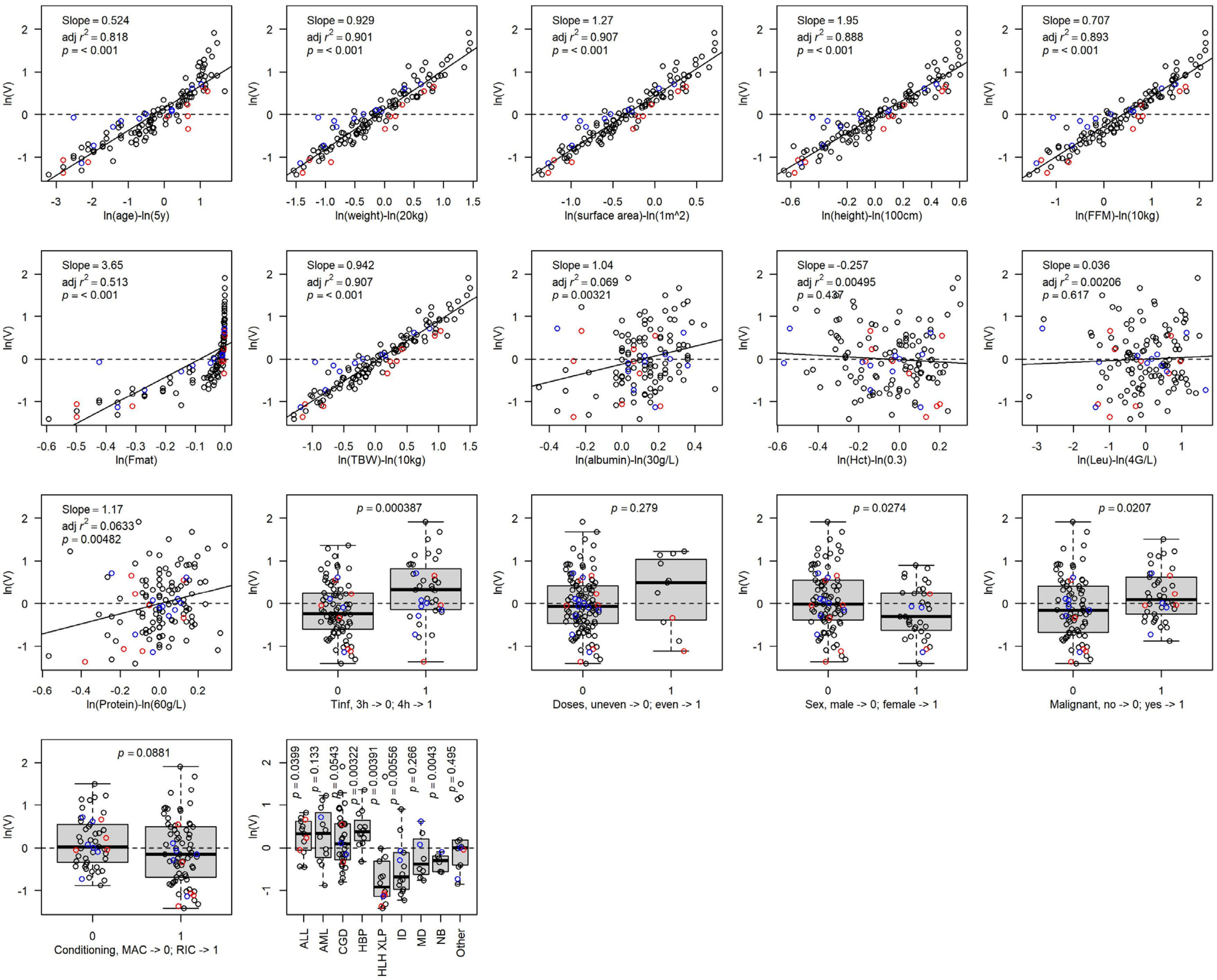

Figure S13. Random effects of ln(V) plotted against potential covariates. Fit with a one-compartment model not including any covariate in the model but including an exponential function of time for the rate constant k (see Equations 1-5 of the main manuscript). Linear regressions and box plots. Circles, individual random effects. Blue, the dose was increased by a factor of > 1.33 after the first AUC calculation. Red, the dose was reduced by a factor of < 0.75 after the first AUC calculation. p of linear regressions (for slope=0), t-tests comparing two factors and t-tests comparing one diagnose group to the combined alternative diagnose groups, respectively. All p values are without correction for multiple comparisons.

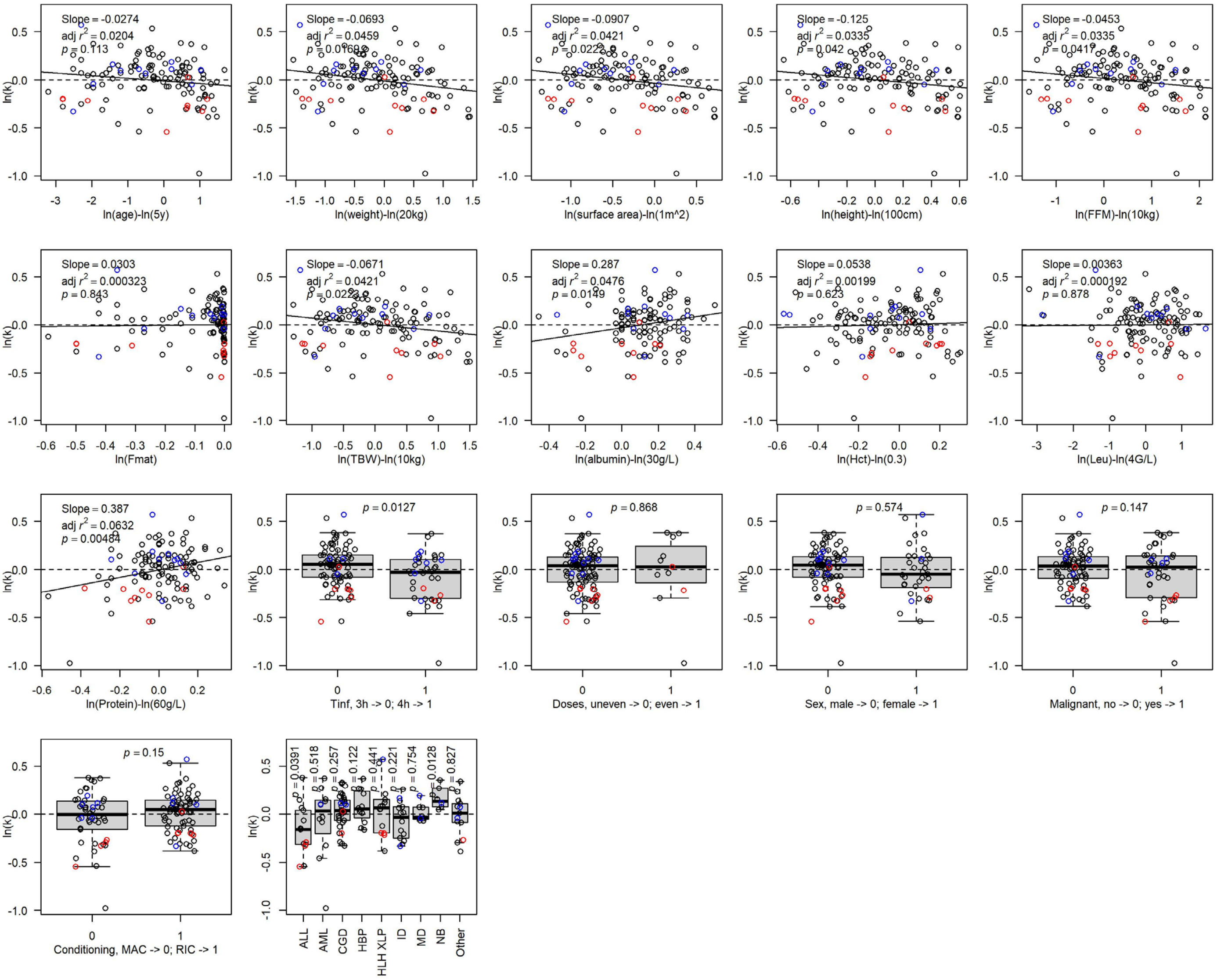

Figure S14. Random effects of ln(k) plotted against potential covariates. Fit with a one-compartment model not including any covariate in the model but including an exponential function of time for the elimination rate constant k (see Equations 1-5 of the main manuscript). Further details, see Figure S13.

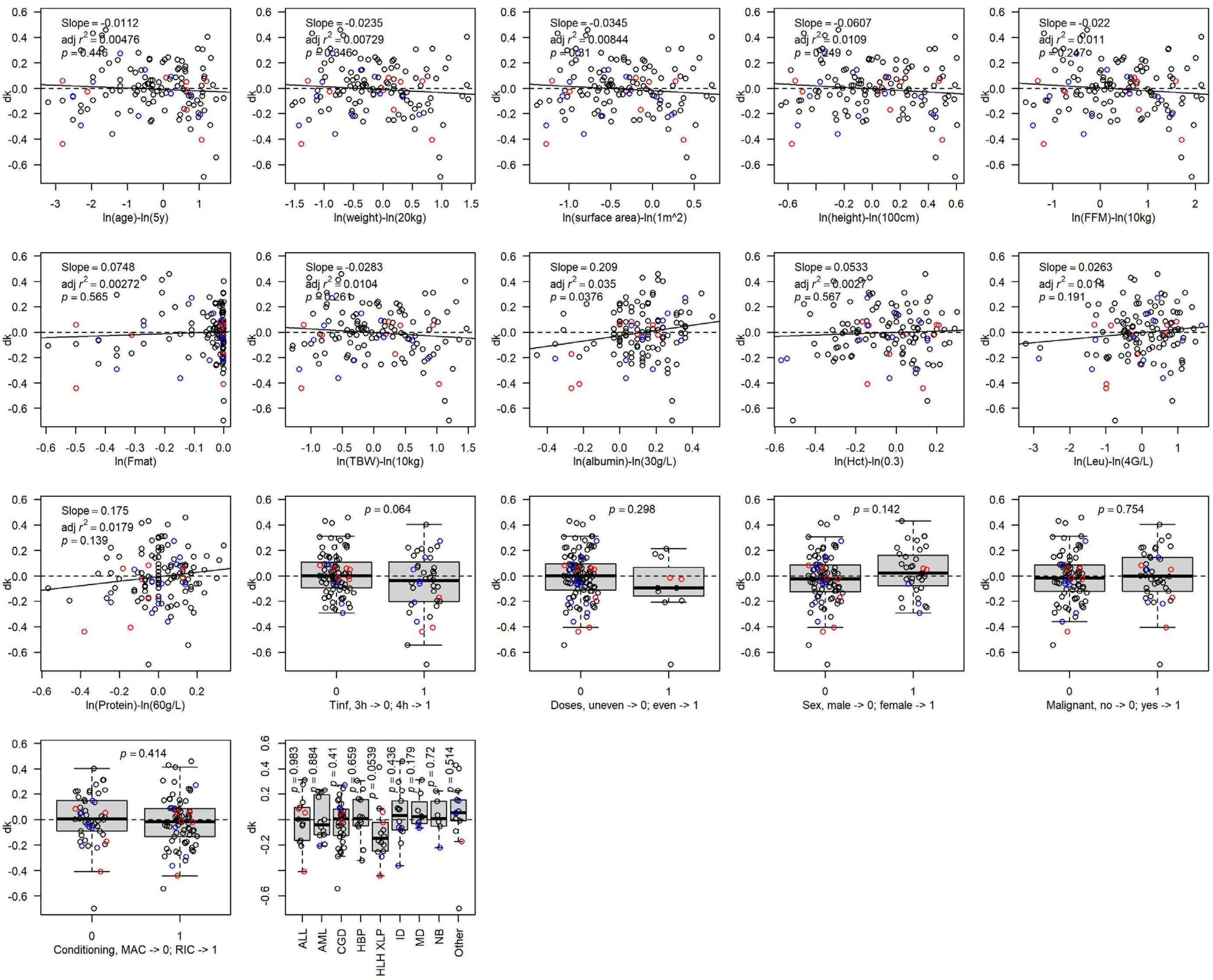

Figure S15. Random effects of d_k plotted against potential covariates. Fit with a one-compartment model not including any covariate in the model. Note, the model did not include random effects for κ_k, the exponent of the exponential function of the change in k *vs* t. Further details, see Figure S13.

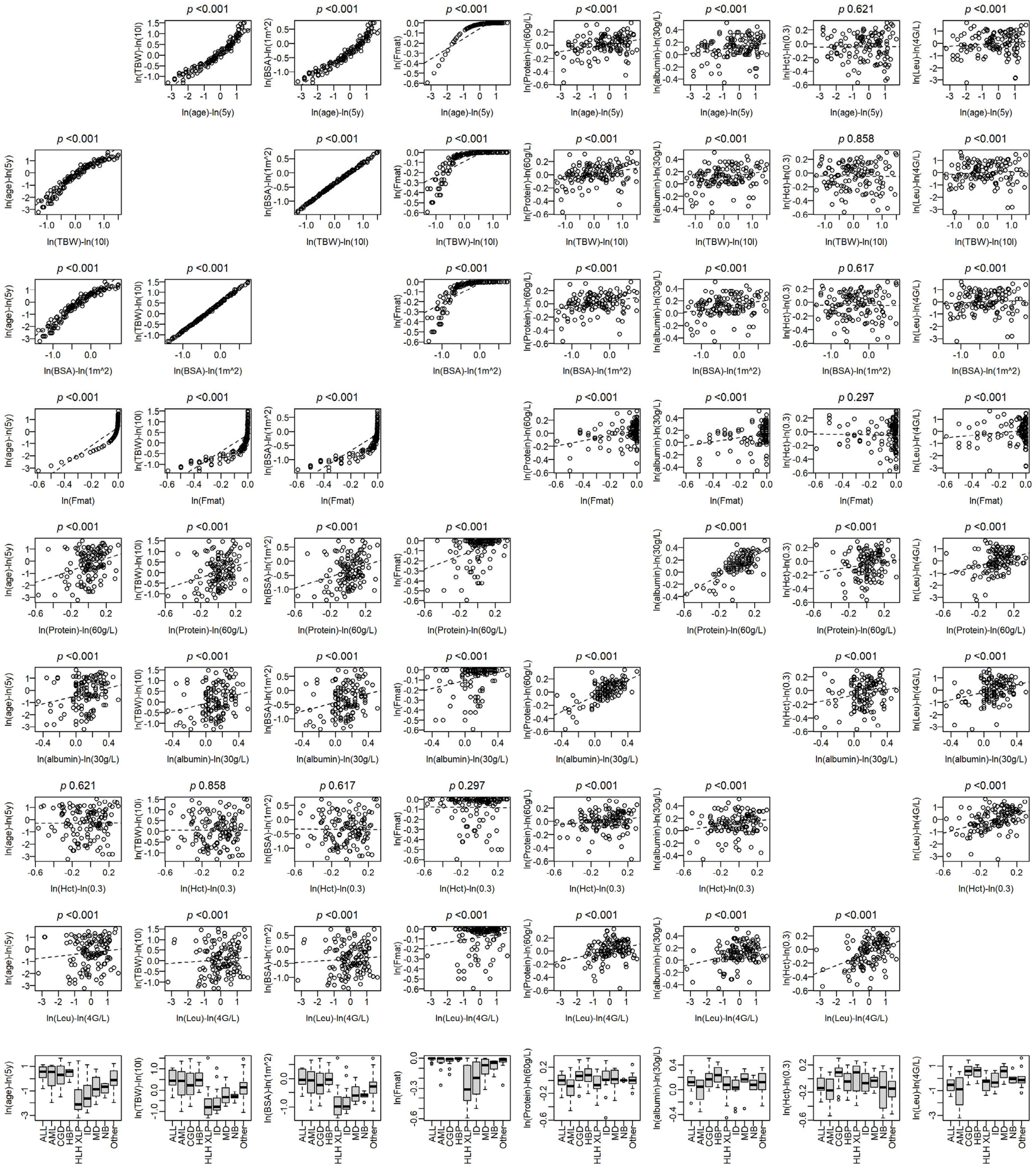

Figure S16. Relations between patient characteristics, with *p*-values of the linear regression slopes above the plots.

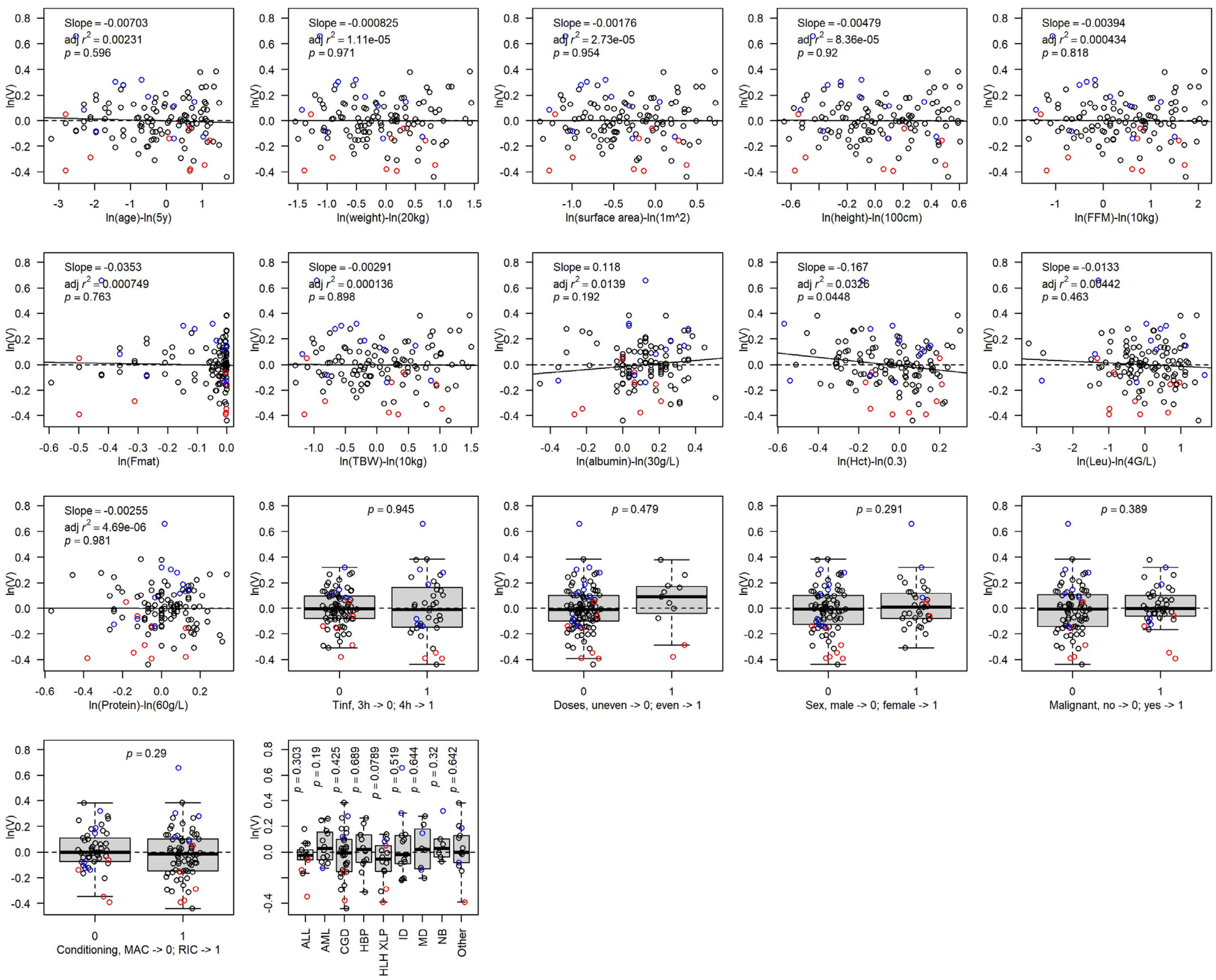

Figure S17. The remaining random effects of ln(V) of the final model (Table 3) plotted against potential covariates. Details, see Figure S13.

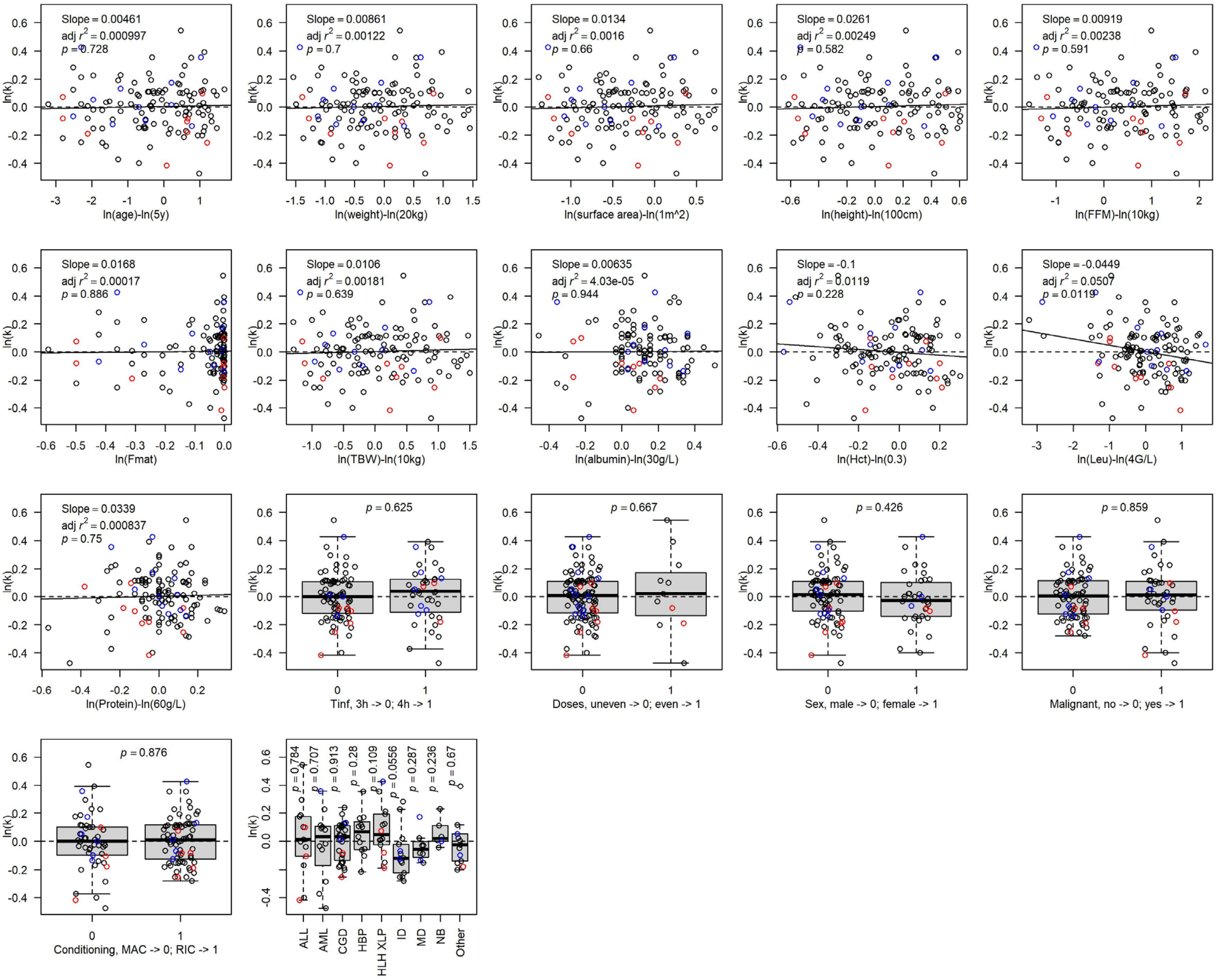

Figure S18. The remaining random effects of ln(k) of the final model (Table 3) plotted against potential covariates. Details, see Figure S13.

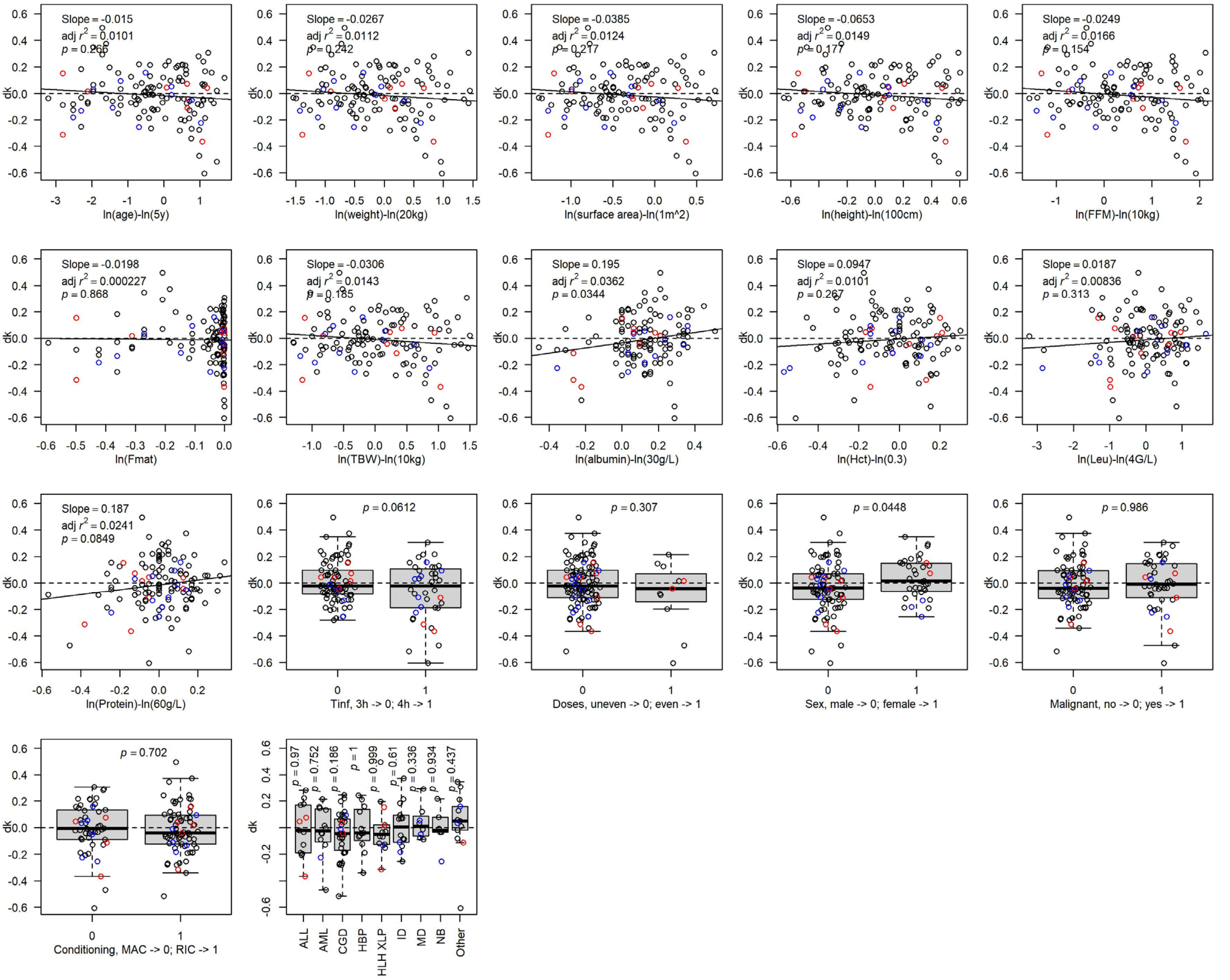

Figure S19. The remaining random effects of d_k of the final model (Table 3) plotted against potential covariates. Details, see Figure S13.

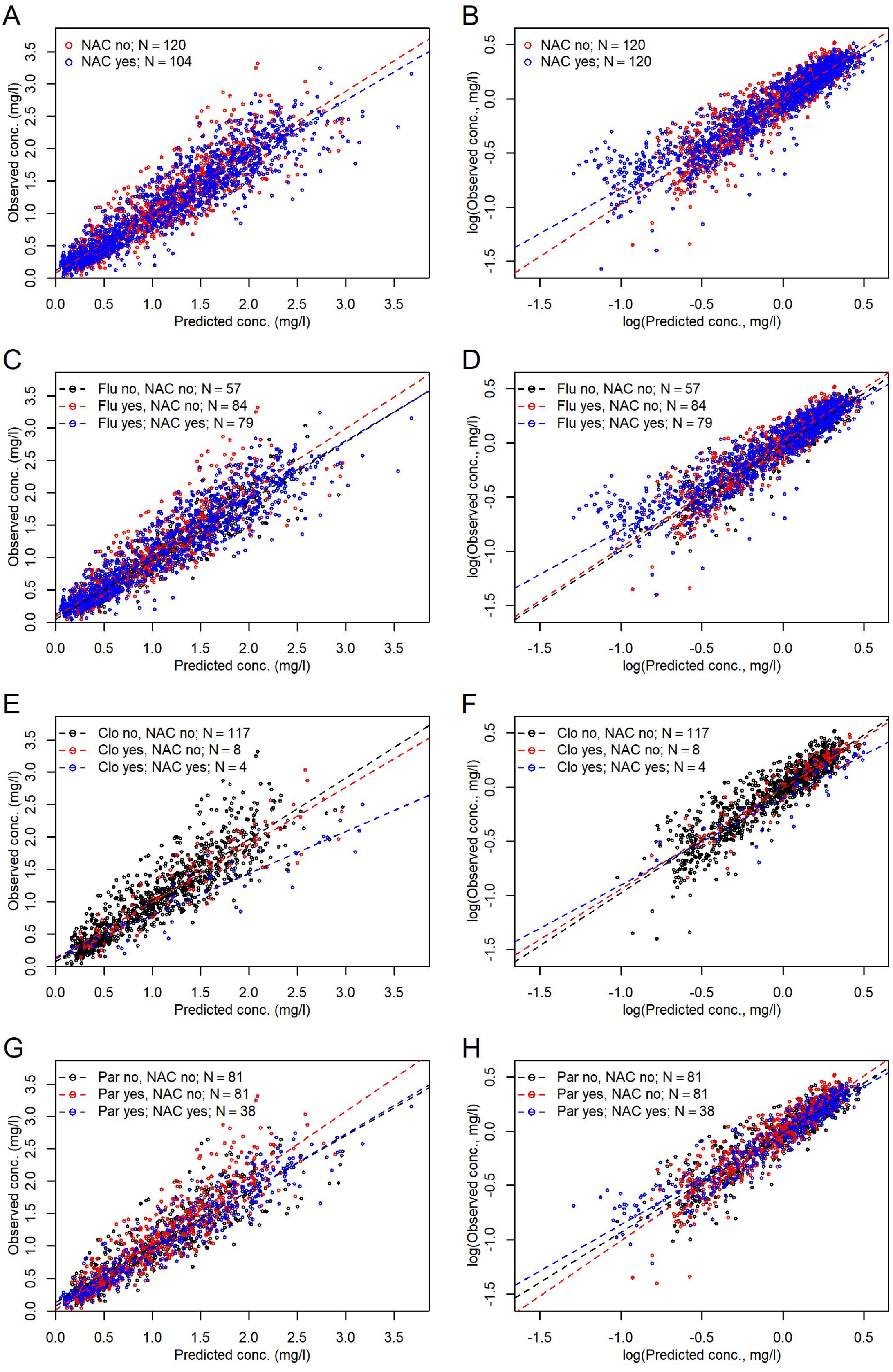

Figure S20. Effects of co-medication on $C(t)$. Plots of observed *vs* predicted $C(t)$ (population level) with linear scales (**A**,**C**,**E**,**G**) and logarithmic scales (**B**,**D**,**F**,**H**). **A**,**B**) NAC. **C**,**D**) Fludarabine. **E**,**F**) Clofarabine. **G**,**H**) Paracetamol. Broken lines, linear regressions. Color codes, see legends in panes.

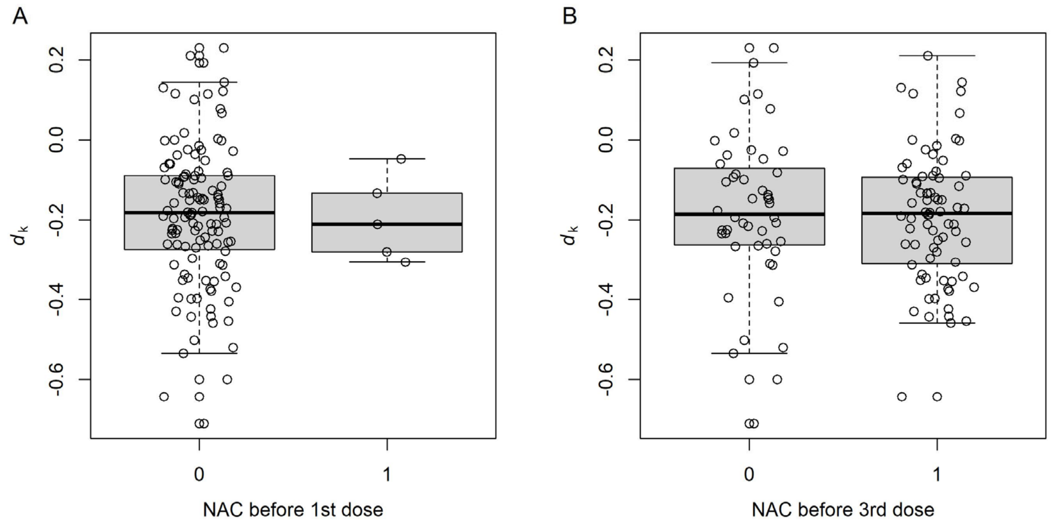

Figure S21. Parameter d_k on subject level for subjects with or without NAC administration within 24h before the 1st (**A**) or 3rd (**B**) busulfan dose (corresponding to doses with $C(t)$ measurements for most patients).

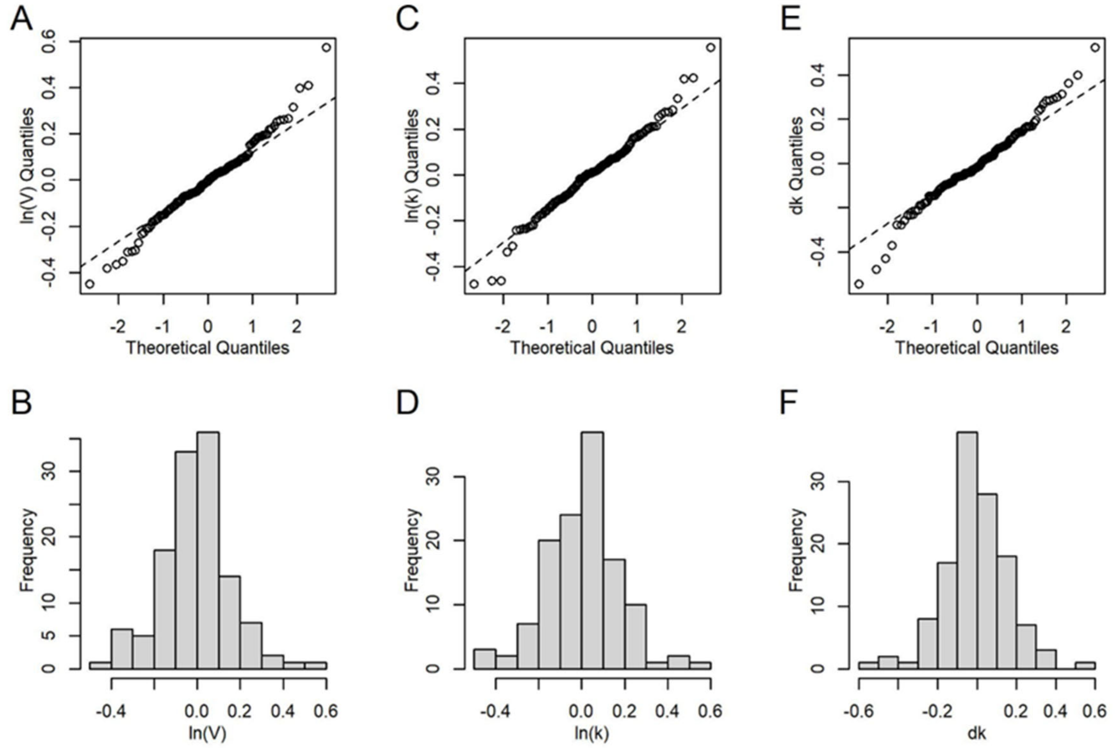

Figure S22. QQnorm plots (**A**,**C**,**D**) and histograms (**B**,**D**,**F**) of the distributions of the remaining random effects of the final model. **A**,**B**) for ln(V); **C**,**D**) for ln(k); **E**,**F**) for d_k. Broken lines, normal distribution. No random effects were included in the model for κ_k.

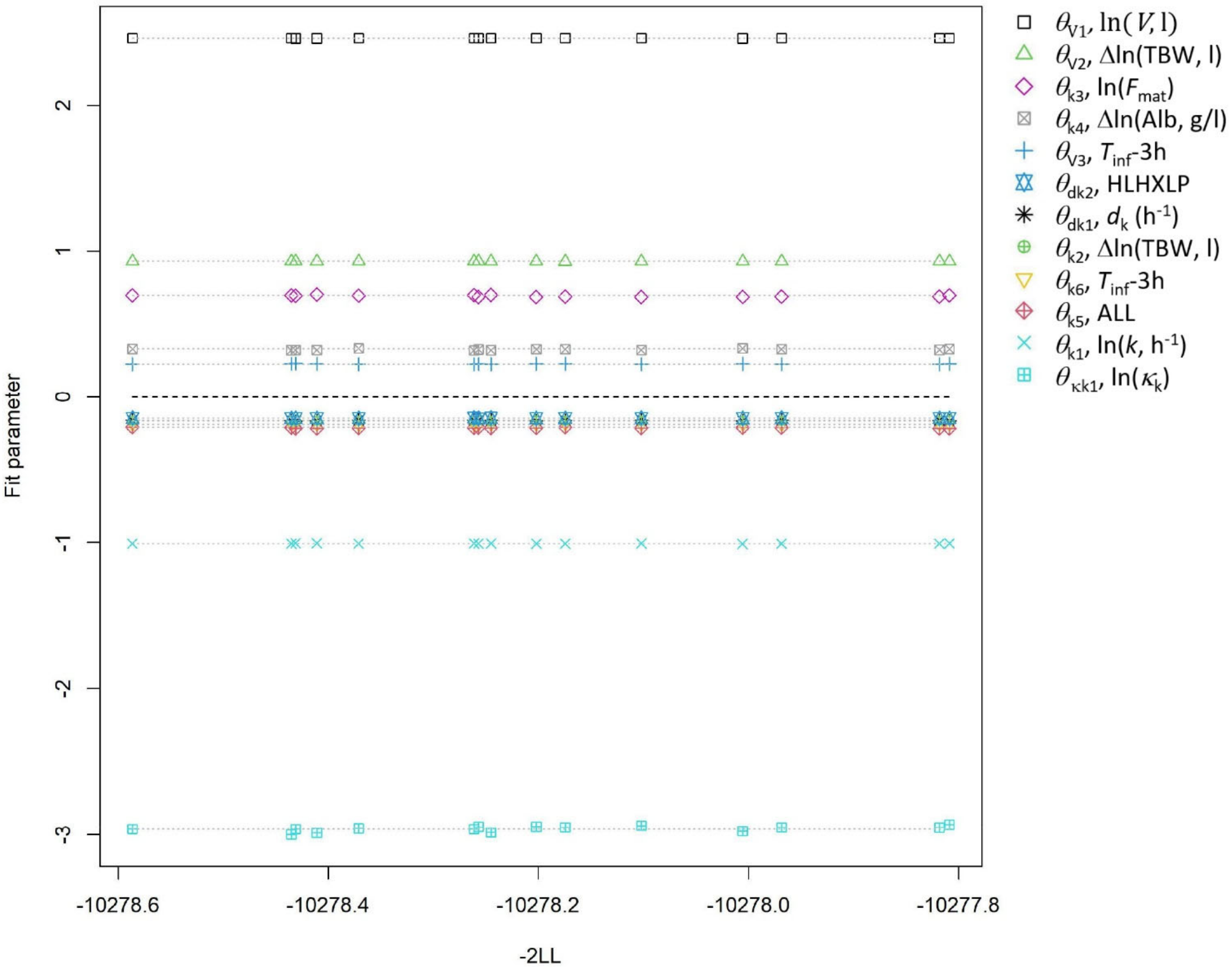

Figure S23. Fit parameters of the final model described in Table 3 from 15 runs plotted against the respective -2LL. Start values were randomly picked from ranges of -1 to +1 of an estimated value based on fits of individual $C(t)$ *vs* t curves (note that the start and fit values are ln(values), except d_k). Start values for the effects of the covariates were 0. Symbols, individual fit values. Broken line at 0; horizontal dotted gray lines, value at lowest -2LL (best fit, transferred to Table 3).

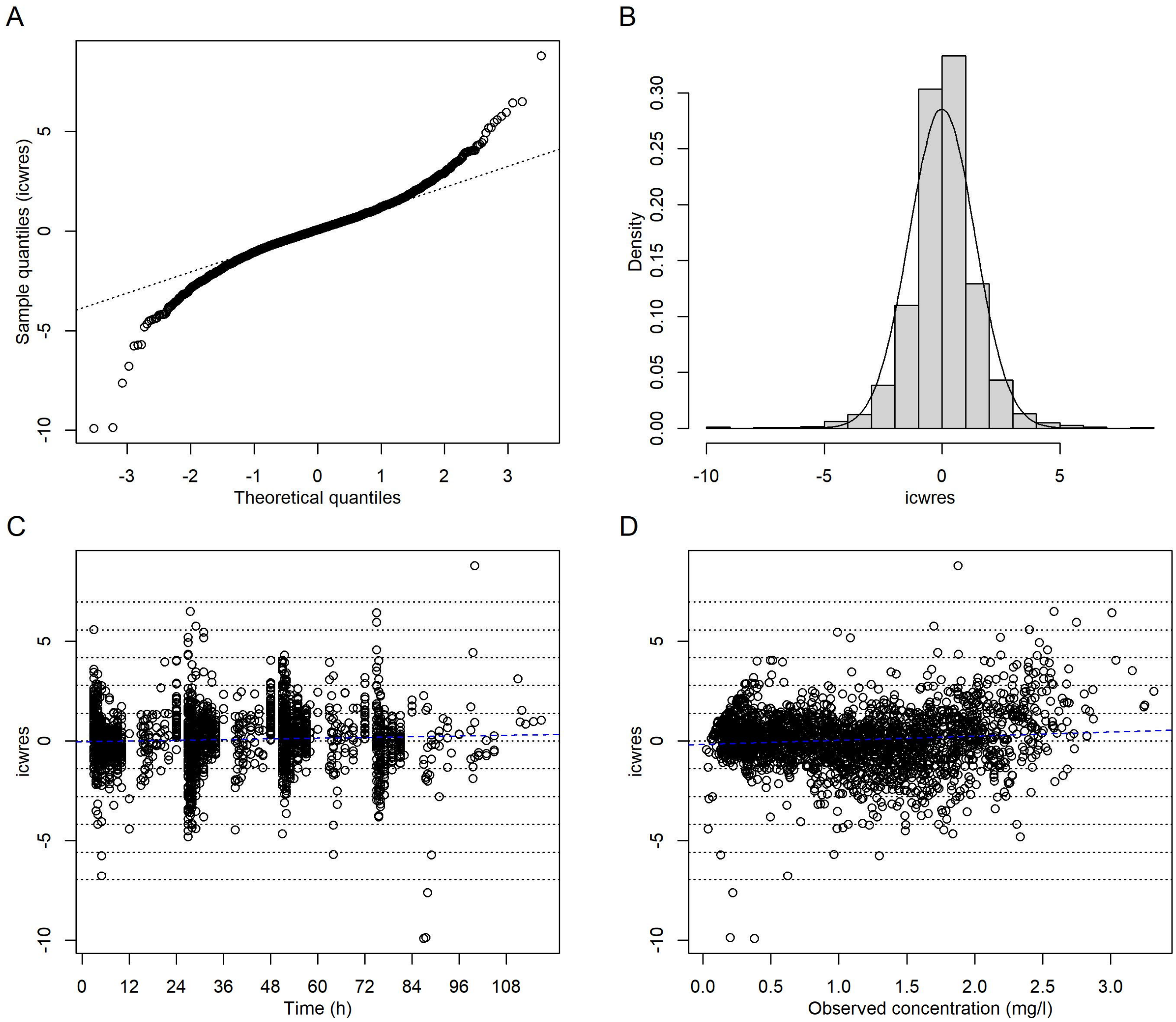

Figure S24. icwres plots for model evaluation. **A**) QQnorm plot. Dotted line, normal distribution for comparison. **B**) Non-parametric distribution of icwres (histogram) and normal distribution (solid line) with SD of icwres. **C**) icwres *vs* t. D) icwres *vs* observed $C(t)$. Dotted horizontal lines in **C** and **D**, multiples of the SD of the icwres. Blue broken lines, linear regressions.

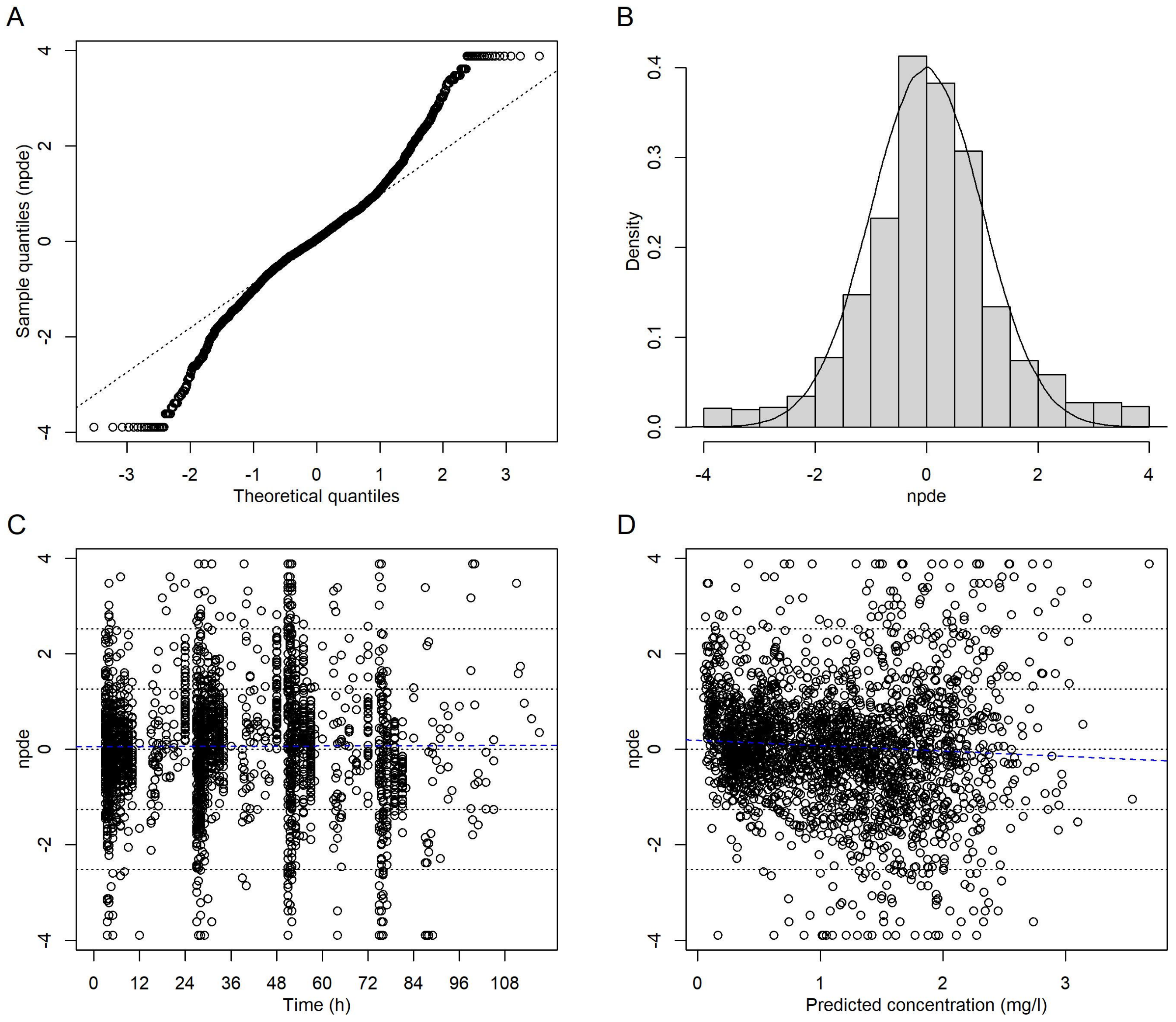

Figure S25. npde plots for model evaluation. **A**) QQnorm plot. Dotted line, normal distribution for comparison. **B**) Non-parametric distribution of npde (histogram) and normal distribution (solid line) with SD = 1. **C**) npde *vs* t. D) npde *vs* predicted $C(t)$. Dotted horizontal lines in **C** and **D**, multiples of the SD of the simulated npde. Blue broken lines, linear regressions.

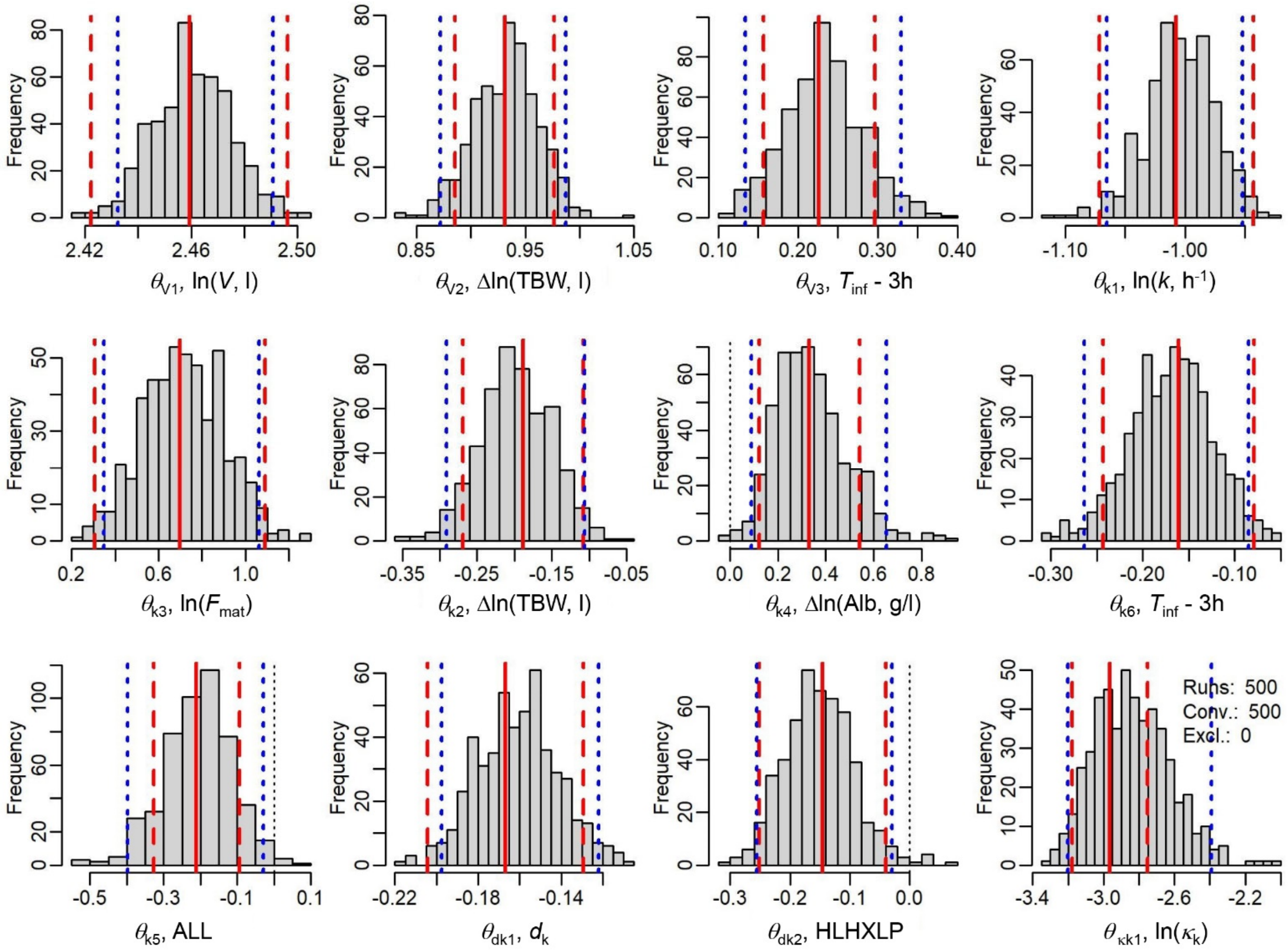

Figure S26. Results from bootstrapping. Distributions of the fit parameters of 500 datasets resampled from the original data set. Data sets had the same size as the original set, meaning that subjects may be included more than once in a data set. Red vertical lines, fit parameters of the full data set (see Table 3). Red broken lines, lower and upper confidence intervals (95%) of the fit parameters of the original data set. Blue dotted lines, confidence intervals (95%) of the fit parameters from the resampled datasets (also indicated in Table 3). Black dotted lines at 0. Insert in last pane, 500 runs (Runs), 500 fits converged (Conv.), 0 fits excluded (Excl.).

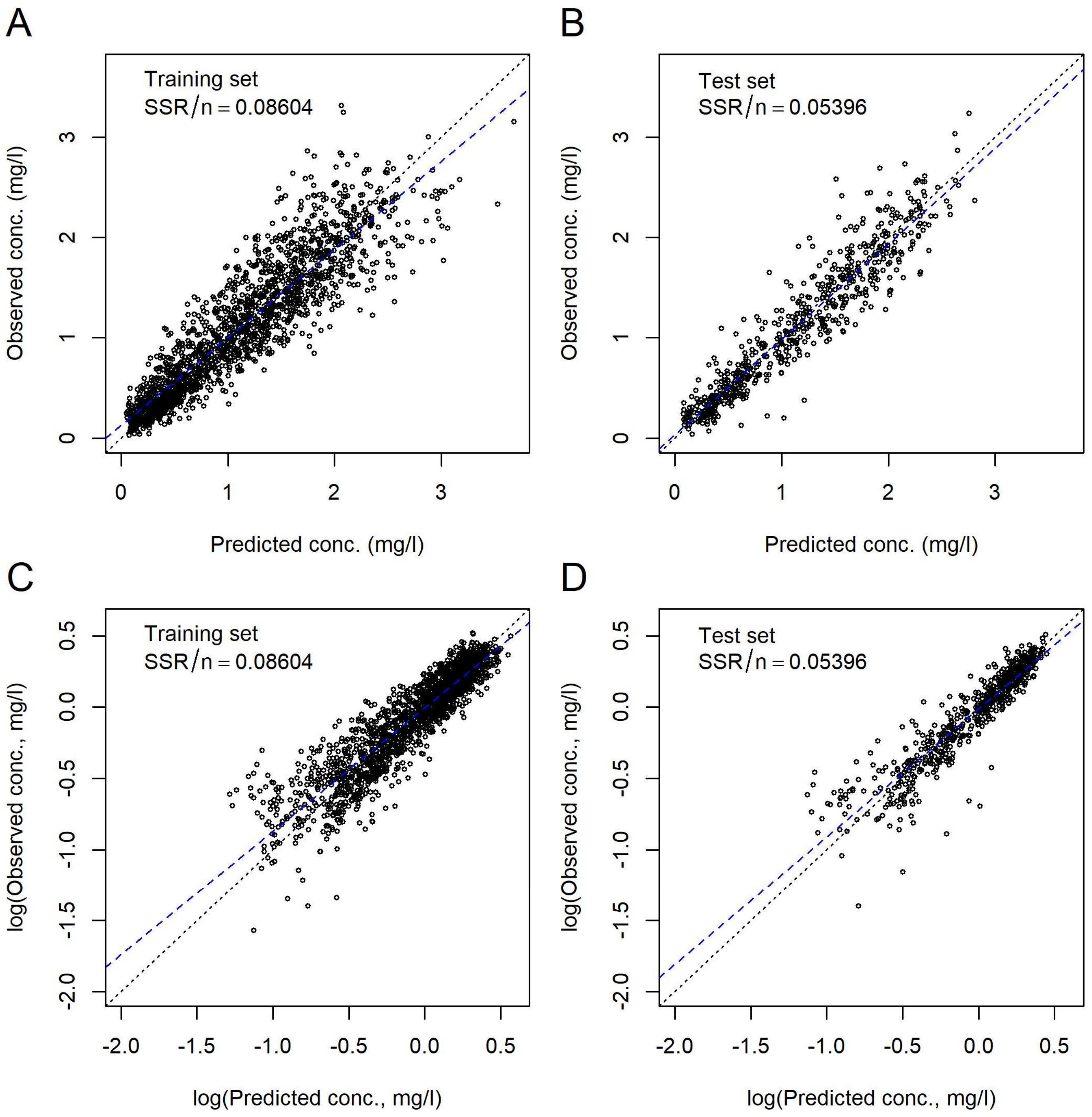

Figure S27. Observed *vs* predicted busulfan $C(t)$ with randomly sampled training and test sets (ratio 75/25). **A**,**C**) Training set; **B**,**D**) test set. **A**,**B**) Linear scales; **C**,**D**) Logarithmic (base 10) scales. SSR/n, sum of squared residues divided by the number of observed $C(t)$ values in the respective set. The fit parameters were similar as for the final model in Table 3: θ_{V1}, 2.458; θ_{V2}, 0.928; θ_{V3}, 0.213; θ_{k1}, -1.016; θ_{k2}, -0.215; θ_{k3}, 0.739; θ_{k4}, 0.350; θ_{k5}, -0.134 (ALL, reduced -2LL only by 2.27); θ_{k4}, -0.146; θ_{dk1}, -0.172; θ_{dk2}, -0.164; $\theta_{\kappa k}$, -2.980. p was <0.05 for all fit parameters. Variances of the random effects were 0.033 (ln(V)), 0.030 (ln(k) and 0.033 (d_k). Dotted black lines, lines of identity. Broken blue lines, linear regressions.
